# Natural and Synthetic LDL-Based Imaging Probes for the Detection of Atherosclerotic Plaques

**DOI:** 10.1021/acsptsci.4c00667

**Published:** 2025-02-04

**Authors:** Alessandro Fracassi, Hui Qiao, Andrew N. Lowell, Jianbo Cao, Jeffrey W. Bode, Hisao Masai, Naoko Yoshizawa-Sugata, Rong Zhou, Yoko Yamakoshi

**Affiliations:** † Department of Chemistry and Applied Biosciences, ETH Zürich, Vladimir-Prelog-Weg 3, Zürich CH8093, Switzerland; ‡ Department of Radiology, Institute for Translational Medicine and Therapeutics, University of Pennsylvania, John Morgan 198, 3620 Hamilton Walk, Philadelphia, Pennsylvania 19104, United States; § Department of Chemistry, Virginia Polytechnic Institute and State University, Davidson Hall, Virginia Tech, 1040 Drillfield Drive, Blacksburg, Virginia 24061, United States; ∥ Department of Basic Medical Sciences, Tokyo Metropolitan Institute of Medical Science, 2-1-6 Kamikitazawa, Setagaya, Tokyo 156-8506, Japan; ⊥ Research Center for Genome & Medical Sciences, Tokyo Metropolitan Institute of Medical Science, 2-1-6 Kamikitazawa, Setagaya, Tokyo 156-8506, Japan

**Keywords:** lipid nanoparticles, imaging probe, KAT ligation, atherosclerosis, MRI, whole-body fluorescence imaging, low-density lipoprotein

## Abstract

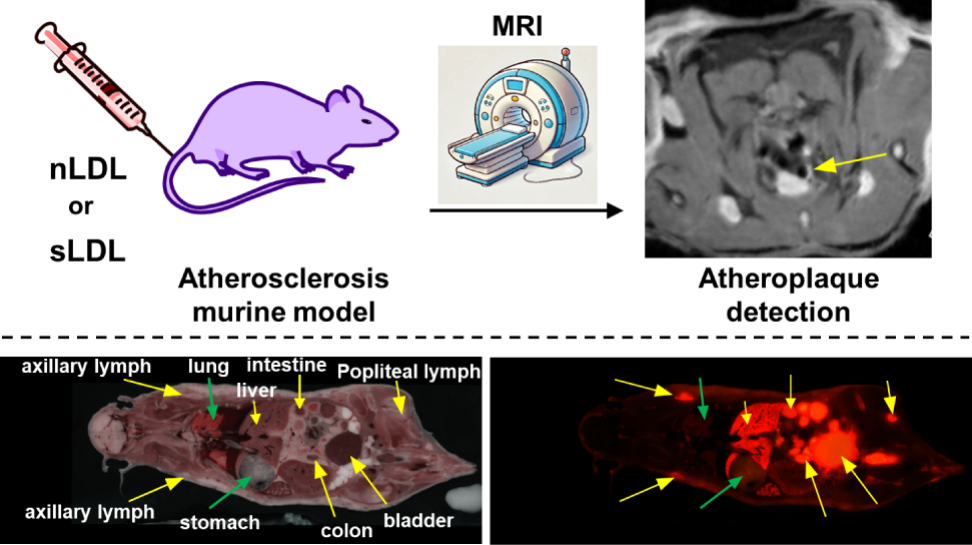

Low-density lipoprotein (LDL) is the primary natural carrier of lipids in the bloodstream and plays a central role in the development of atherosclerosis. By leveraging LDL’s natural tendency to accumulate at sites of plaque formation, LDL can be employed as a carrier to selectively deliver the imaging probes to efficiently detect atherosclerotic plaques. In our previous studies, we reported several LDL-based magnetic resonance imaging contrast agents (MRI-CAs) formed by modifying natural LDL (nLDL) or developing LDL-mimetic (synthetic LDL, sLDL) from lipid nanoparticles (LNPs) utilizing chemical reactions on the nanoparticle surface, including preliminary MRI tests. In this study, we report the *in vivo* biological functionality of these LDLs (both nLDL and sLDL)-based Gd(III)-based contrast agents (GBCAs) by conducting detailed *in vivo* studies on two types of atherosclerosis murine models, namely, *apoE*^*–/–*^ and *LDLr*^*–/–*^. We provide more comprehensive MRI data accompanied by *ex vivo* results, including microscopic analysis of aorta segments for LDL accumulation and whole-body cryoVIZ analysis for biodistribution of the probe. We also tested *in vitro* cellular internalization of sLDL on two cell lines (RAW 264.7 and THP-1), which are derived from macrophages and monocytes, respectively, in order to observe sLDL uptake by macrophages, which are often present at the vulnerable types of atherosclerotic plaques. In conclusion, our current study demonstrates that modified LDLs—both nLDL and sLDL—facilitate MRI detection of atheroplaques by efficient uptake by macrophages. Taken together with the high loading capacity of Gd(III)-chelate molecules on LDL, especially sLDL, the LDL-based MRI contrast agents reported here hold significant potential for the early detection of atherosclerosis, including vulnerable ones, and should be useful for preventive diagnosis strategies.

Atherosclerosis is an inflammatory disease of the arteries, generally characterized by the deposition of fatty materials on their inner arterial wall.^1^ The progression of atherosclerosis is typically assessed by evaluating the degree of stenosis (*i.e.*, narrowing the lumen of an artery).^2^ However, stenosis does not reveal the risk of plaque rupture, which often results in lethal cardiovascular events such as stroke and heart attack.^3,4^ Sensitive imaging probes, which provide high-resolution images of vulnerable atheroplaques, are in high demand. Among various imaging modalities, magnetic resonance imaging (MRI)^5^ is recognized as a suitable imaging method for atherosclerosis, since it provides high-spatial-resolution images with excellent soft tissue contrast in a noninvasive manner with a lack of ionizing radiation. Especially when targeted delivery of MRI contrast agent (MRI-CA) is possible, MRI has a significant potential to precisely characterize the plaque size and morphology, and to further estimate the fibrous cap thickness, a critical factor for plaque rupture events.^6^ Given the well-studied role of lipoproteins (low-density lipoprotein (LDL) and high-density lipoprotein (HDL)) involved in driving plaque formation,^7^ several groups have designed lipoprotein-based MRI-CA and achieved the detection of plaques. These overcome the limitation of commercially available small molecule Gd(III)-chelates, which do not significantly accumulate in atheroplaques and, hence, are not applicable for the detection of plaques *in vivo*.^8−12^

In our previous work, we have initially focused on the use of natural LDL (nLDL) as a delivery vehicle of the Gd(III)-chelates to atheroplaques.^12,13^ LDL plays a central role in the development and progression of atherosclerosis, suggesting its great potential as a target vehicle to atherosclerosis.^14^ While the use of nLDL for drug delivery to cancers has been documented,^15^ studies utilizing LDL for atherosclerosis diagnosis and treatment remain limited. After our initial study, which confirmed the ability of nLDL to deliver MRI-CA to the plaques, resulting in sufficient enhancement *in vivo*, we have shifted our focus to the development of a synthetic version of LDL (sLDL), which can be produced from commercially available lipids. The nLDL requires the human blood from donors and may cause infectious side effect when used clinically. In contrast, sLDL cost less to prepare and has no risk of infection. While the synthetic HDL using apoA1 mimetics has been reported,^16−18^ LDL-mimetic nanoparticles, sLDL, were not well studied. This was mainly due to the difficulties in handling the apolipoprotein (apoB100, 512 kDa),^19,20^ which is much larger and insoluble in comparison to the apoA1 (12.76 kDa), an apolipoprotein for HDL. Although challenging, considering LDL’s critical role as the primary carrier of cholesterol and lipids in the human bloodstream, sLDL-based delivery systems present a significant opportunity warranting further investigations in the field of cardiovascular diagnostics and therapeutics. Further advantages of sLDL include reduced immunogenicity, better stability, controlled composition with consistent batch quality, elimination of contamination risks, and reduced costs.

Our previously reported nLDL-based Gd(III)-based contrast agents (GBCAs), prepared from nLDL by intercalation of a Gd(III)-chelate derivative, successfully provided MR-active nLDL (**nLDL-Gd**) with a significantly high payload of Gd(III)-chelate (>200 Gd^3+^ per LDL particle). This resulted in a higher relaxivity value per Gd^3+^ (*r*_1_ = 20 s^–1^·mM^–1^ per Gd^3+^) in comparison to the small molecule GBCA (Gd(III)-DOTA, *r*_1_ = ca. 4–5 s^–1^·mM^–1^ per Gd^3+^), presumably due to slower tumbling of GBCA when attached to the larger nanocarriers.^13^ The sLDL-based MRI-CA was prepared from a lipid nanoparticle made from a mixture of commercially available lipids and synthetic materials.^21,22^ By building on the previous study by Forte and coworkers on LDL-mimetic nanoparticles, we used a mixture of lipids (phosphatidylcholine, triolein, and cholesteryl oleate).^23,24^ We added a synthetic lipid with a terminal potassium acyltrifluoroborate (KAT) group to prepare a versatile core lipid nanoparticle (**LNP-KAT**). This KAT group was recently developed as a convenient functional group that reacts with hydroxylamine (HA) derivatives as ligation partners in a chemoselective and bioorthogonal manner to form natural amide bonds.^25^ We adopted this relatively new reaction for the LNP surface functionalization, which should be performed under aqueous physiological conditions to avoid damage of LNP and biomolecules. As we expected, core **LNP-KAT** was prepared with a homogeneous distribution of diameter (with a mean of ca. 50 nm) having a sufficient amount of polar KAT groups displayed on the surface. Subsequent on-surface KAT ligation of the **LNP-KAT** core with HA derivatives of apoB100-mimetic peptide and Gd(III)-chelate successfully provided sLDL-based MRI-CA (**sLDL-Gd**) functionalized with apoB100-mimetic peptide. We determined that they had a high payload of Gd^3+^ (2800 Gd atoms per sLDL), providing a suitable *r*_1_ relaxivity (22.0 s^–1^·mM^–1^ per Gd^3+^) without aggregation.

In our previous studies,^13,22^ we documented the preparation and detailed characterization of nLDL- and sLDL-based MRI-CA with a focus on surface chemistry, and only preliminary biological data were included. In the present study, we observed comprehensive biological studies to evaluate nLDL- and sLDL-based MRI-CA on their ability to target atheroplaques by *in vivo* MRI in combination with *ex vivo* studies using two genetically engineered atherosclerosis mouse models, *apoE*^*–/–*^ and *LDLr*^*–/–*^. Specifically, using **nLDL-Gd** and **nLDL-DiI** (commercially available dialkyl indocarbocyanine-labeled nLDL), we report the accumulation of **nLDL-Gd** to the aortic tissues of *apoE*^*–/–*^ and *LDLr*^*–/–*^ measured by ICP-MS analyses for Gd^3+^ contents and localization of **nLDL-DiI** within the atheroplaques by fluorescence microscopy together with immunohistochemistry (IHC) for both mouse models. For the study of sLDL, we used **sLDL-Gd** and **sLDL-GdR** (rhodamine-loaded **sLDL-Gd**) and performed MRI on *apoE*^*–/–*^ and *LDLr*^*–/–*^ in combination with ICP-MS analysis of the aorta and high-resolution whole-body cryosectioning and imaging for the evaluation of the biodistribution of the sLDL-based probes.

## Experimental Section

### Preparation of nLDL with a GBCA (nLDL-Gd)

**Figure 1:**
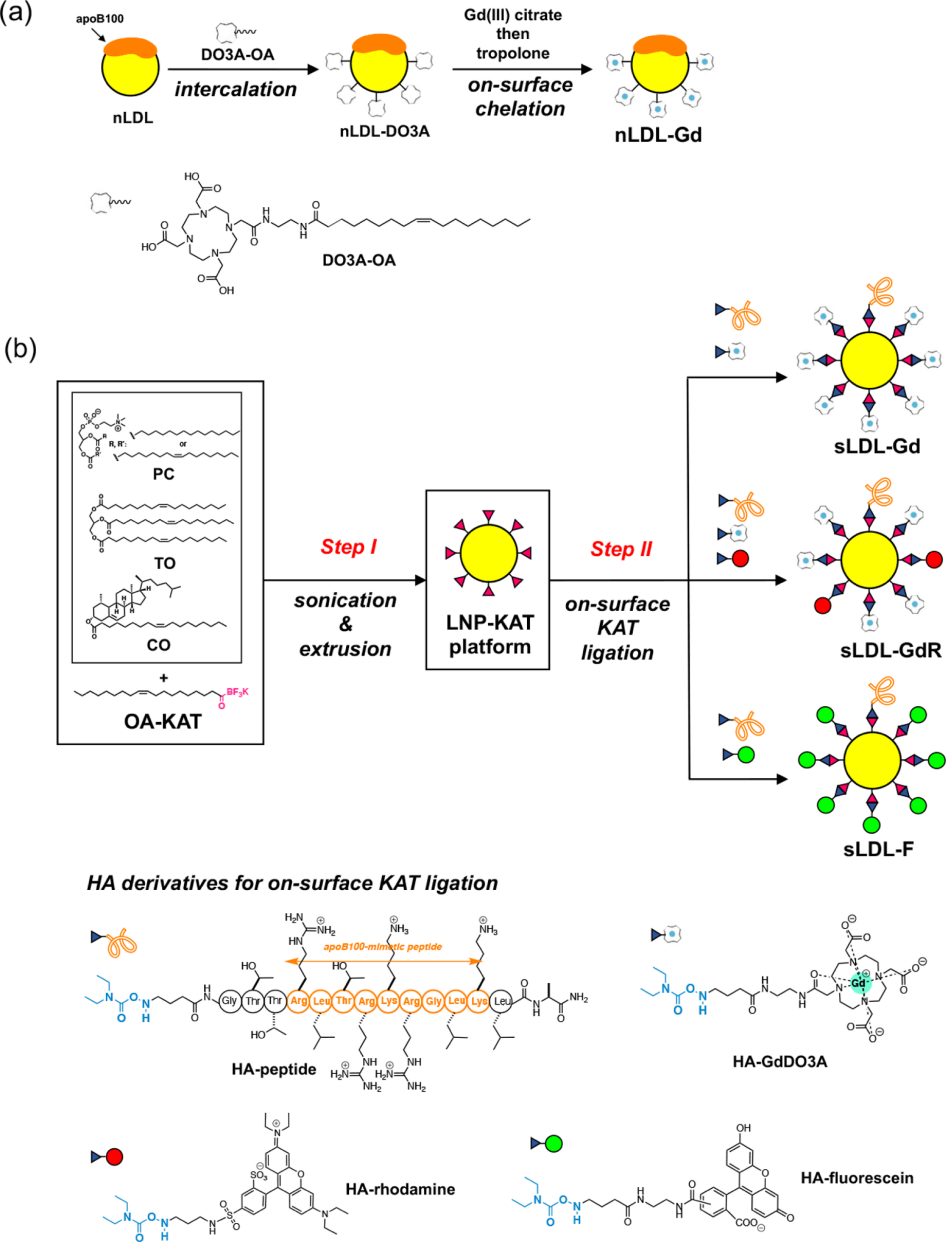
Schematic illustration for the preparation of LDL-based nanoparticles. (a) Preparation of **nLDL-Gd** from nLDL by intercalation of **DO3A-OA** and subsequent Gd^3+^ chelation. (b) Preparation of sLDL-based probes (**sLDL-Gd**, **sLDL-GdR**, and **sLDL-F**) *via***LNP-KAT** platform. The **LNP-KAT** was prepared by sonication and extrusion of a mixture of lipids (*Step I*), followed by on-surface KAT ligation with HA derivatives (*Step II*) of apoB100-mimetic peptide (**HA-peptide**), Gd(III)-chelates (**HA-GdDO3A**), and fluorophores (**HA-rhodamine** and **HA-fluorescein**).

The preparation of **nLDL-Gd** was performed according to the previous report (Figure 1a).^13^ Briefly, nLDL was isolated from the blood of healthy human donors by centrifugation in the laboratory of Prof. Lund-Katz at the Children’s Hospital of Philadelphia^26^ and subsequently coincubated with an oleic acid derivative of DO3A (**DO3A-OA**, Figure 1a) in PBS(−). The obtained nLDL dispersion was subjected to the on-surface Gd^3+^ chelation reaction with gadolinium citrate (Figure 1a). Unbound free Gd^3+^ ions were removed by the addition of tropolone, which forms an insoluble precipitate upon Gd^3+^ chelation.

### Preparation of the LNP-KAT Platform

The LNP-KAT particles were prepared according to our previous report.^22^ Briefly, a solution of a mixture of three commercially available lipid components (Figure 1b, *Step I*), phosphatidylcholine (**PC**, 44.3 μmol), triolein (**TO**, 33 μmol), and cholesteryl oleate (**CO**, 16.5 μmol) in 90 mL of CHCl_3_–MeOH–acetone (2:1:1, v/v), was mixed with a solution of oleic acid KAT derivative (**OA-KAT**) (5 μmol) in 30 mL of MeOH–acetone (1:1, v/v). The solvents were then slowly evaporated under vacuum to provide a lipid thin film at the bottom of the round-bottom flask. To this film, 4 mL of Tris-HCl buffer (10 mM, pH 8.0, containing 10 mM KF and 1 mg·mL^–1^ BHT) was added, sonicated, extruded with two stacked polycarbonate membrane filters (pore sizes: 0.05 and 0.1 μm), and filtered (0.45 μm membrane) (*Step I* in Figure 1b). The filtrate was washed with Tris-HCl buffer by spin filtration (50 kDa MW cutoff) to remove free lipids, which were not incorporated in the particles, to provide 4 mL of **LNP-KAT** (5%) particle dispersion in Tris-HCl buffer (**LNP-KAT** platform in Figure 1b). The **LNP-KAT** dispersion was stored at 4 °C. Similarly, **LNP-KAT** (10%) particle dispersion (4 mL) was prepared using a lipid mixture of **PC** (40 μmol), **TO** (33 μmol), **CO** (17 μmol), and **OA-KAT** (10 μmol).

### Preparation of sLDL with GdDO3A (sLDL-Gd)

The hydroxylamine (HA) derivatives of apoB100-mimetic peptide (**HA-peptide**, Figure 1b) and GdDO3A (**HA-GdDO3A**, Figure 1b) were synthesized according to the previous report.^22^ To a solution of a mixture of **HA-GdDO3A** (135 μM) and **HA-peptide** (15 μM) in 10 mM phosphate buffer (pH 5.8, containing 10 mM KF) (16 mL), 0.1 M HCl (100 μL) and a dispersion of **LNP-KAT** (5%) in Tris-HCl buffer (pH 8.0) (4 mL) were added and stirred overnight at room temperature (Figure 1b, *Step II*). Subsequently, the reaction mixture was subjected to spin filtration and washed with PBS(−) to remove unreacted HA derivatives.

### Preparation of sLDL with GdDO3A and Rhodamine (sLDL-GdR)

A HA derivative of rhodamine (**HA-rhodamine**, Figure 1b) was synthesized according to the previous report.^22^ The **sLDL-GdR** was prepared using **LNP-KAT** (10%) in a similar manner to the preparation of **sLDL-Gd** above.

### Preparation of sLDL with Fluorescein (sLDL-F)

A hydroxylamine (HA) derivative of fluorescein (**HA-fluorescein**, Figure 1b) was synthesized according to the previous reports.^21,22^ The **sLDL-F** was prepared using **LNP-KAT** (10%) in a similar manner to the preparation of **sLDL-Gd** above.

### Mouse Models

All animal procedures were approved by the Institutional Animal Care and Use Committee (IACUC) of the University of Pennsylvania. Two types of atherosclerotic mouse models, *apoE*^*–/–*^ and *LDLr*^*–/–*^, were used. The *apoE*^*–/–*^ and *LDLr*^*–/–*^ mice (7 weeks old, male) were purchased from Jackson Laboratory (Bar Harbor, ME, USA). Upon arrival, the mice were acclimated for 1 week (at the age of 8 weeks) at the housing facility managed by University Laboratory Animal Resources (ULAR) before starting to receive the high-fat diet (HFD, consisting of 42% kcal from fat, Teklad #88137), which was continued for 8 weeks. The housing facility was dedicated to small rodents with 12-h light cycle, temperature control, and environmental enrichment such as nesting material, which are standard for all ULAR facilities.

To test whether the **nLDL-Gd** or **sLDL-Gd** can visualize the atheroplaques by MRI, we used only male mice because mice are relatively resistant to atherosclerosis even with an HFD compared to humans, and male mice are more likely to develop atheroplaques than female mice. Upon completion of the HFD, the mice were approximately 16 weeks old and were randomly assigned to the experimental groups (receiving **nLDL-Gd** or **sLDL-Gd**). Since we were testing different types of nanoparticle formulations without prior data to prospectively decide the number of mice required, we included 3–4 mice in each experimental group for *invivo* MRI and 2–3 mice for *ex vivo* fluorescence imaging. The number of mice used in each experiment is reported in the figure captions for clarity. The volumes of injection were about 250 μL per mouse.

### *In Vivo* Accumulation Test of nLDL-Gd in Atherosclerotic Mouse Models and Control Mice

Two types of atherosclerosis mouse models (*apoE*^–/–^ and *LDLr*^–/–^), purchased from the Jackson Laboratory and fed with high-fat diet (HFD) for 5 months, were injected with **nLDL-Gd***via* a catheter placed in the mouse tail vein under anesthesia along with a control mouse model (C56*L*/6j). The dissected aortas were subjected to *ex vivo* ICP-MS analysis, which was performed in the Toxicology Core Lab at the New Bolton Center of Veterinary Hospital of the University of Pennsylvania.

### *In Vivo* MR Imaging with sLDL-Gd and sLDL-GdR on Atherosclerotic Mouse Models

Both *apoE*^–/–^ and *LDLr*^–/–^ mouse models were used for *in vivo* MRI. After being on an HFD for 5 months, the mice were injected with **sLDL-Gd** or **sLDL-GdR** particles *via* a catheter placed in the mouse tail vein under anesthesia. All MRIs were performed on a 9.4 T horizontal bore MR spectrometer (DirectDrive, Agilent, Palo Alto, CA, USA) equipped with a 12 cm (ID) gradient coil. The mouse was positioned prone in a quadrature volume radio frequency (RF) coil (ID = 3.5 cm, length = 8 cm, m2m imaging/Polarean) tuned to the ^1^H resonance frequency (400 MHz). During imaging, the mouse was sedated with 0.8–1% isoflurane mixed with air (flow rate = 1 L min^–1^). Electrocardiogram (ECG), respiration, and core temperature of the mouse were monitored by using an MRI-compatible vital sign monitoring system (SA Inc., Long Island, NY, USA). The rectal temperature was maintained at 37 ± 0.2 °C by a feedback loop that turns on/off warm air directed into the magnet bore. Scout images were acquired to capture the aortic arch with the left common carotid (LC), left subclavian (LS), and brachiocephalic (BA) arteries. Subsequently, the image plane was placed to cut through the three aortic branching points. This image plane was used to acquire the “white blood” (WB) images, in which the blood signal in the aorta lumen is not suppressed, as well as the “black blood” (BB) images, where the blood signal is suppressed (thus black). To obtain the WB image, an ECG-gated multislice gradient echo sequence (TR = 1 heartbeat, about 120 ms, TE = 2.4 ms) was employed with FOV = 26 × 25 mm, matrix size = 192 × 128, slice thickness = 0.8 mm. To obtain BB images, ECG-gated multislice FLASH (fast imaging with low angle shot) (TR = 76 ms, TE = 0.97 ms, FA = 40 degrees) was applied to the same slice. Due to the long interval between MRI sessions, we relied on the unique anatomy of the aortic arch, its branching points, and other thoracic arteries in the imaging plane for comparison of pre- and postinjection images. For images acquired after **sLDL-Gd** or **sLDL-GdR** injection, the BB MRI protocol was further combined with fat suppression.

### *Ex Vivo* Analysis of Atherosclerotic Mouse Models Injected with nLDL-DiI

After being on an HFD for 5 months, *apoE*^–/–^ and *LDLr*^–/–^ mice were injected with 0.3 mL (60 μg) of commercial **nLDL-DiI** (BT-904, Biomedical Technologies Inc., Stoughton, MA, USA) and euthanized 72 h after injection. Thirty minutes before the injection of **nLDL-DiI**, mice were injected with LDL (∼50–100 μg in 0.1 mL) to saturate LDL receptors. The entire aorta was dissected from each mouse. One aorta was placed in IVIS imaging system to capture a fluorescent image (excitation wavelength = 500 nm) overlaid on a white light image of the aorta. The other collected aortas were embedded in Tissue-Tek Optimal Cutting Temperature (O.C.T.) solution (Sakura Finetek USA, Inc., Torrance, CA, USA), and cryosections (10 μm thick) were cut. Sections were subjected to immunohistochemistry (IHC) with CD68 antibody for the visualization of macrophages, and the adjacent sections were hematoxylin and eosin (H&E) stained for anatomies.

### Biodistribution of sLDL-GdR by *Ex Vivo* Whole-Body Cryo-Imaging of Atherosclerotic Mouse Mode

After being on an HFD for 5 months, *apoE*^–/–^ mice were injected with **sLDL-GdR** particles under anesthesia *via* a catheter placed in the mouse tail vein. At 48 h postinjection, the mice were imaged by MRI and then euthanized. Immediately after euthanasia, the mice were embedded in O. C. T. solution and frozen in liquid nitrogen before being transferred to BioInVision Inc. (Cleveland, OH, USA). Concomitant cryosectioning and imaging were performed using an automated microtome-blockface episcopic imaging system (CryoViz, BioInvision Inc.), which allows three-dimensional, high-resolution brightfield and fluorescence imaging of macroscopic specimens. The first mouse was sectioned from dorsal to ventral (coronal view) with a section thickness of 40 μm and an in-plane resolution of 10 μm × 10 μm, which were applied to the entire body. The second mouse was sectioned along the transverse plane (axial view) with the same section thickness specified for the first mouse.

### *In Vitro* Cellular Assay for Cell Culture Studies with sLDL

Two types of cell lines, THP-1 (human monocyte cell line) and RAW 264.7 (mouse macrophage cell line, a gift from Dr. Irie at the Tokyo Metropolitan Institute of Medical Science), were used for the *in vitro* internalization test of sLDL. THP-1 cells were cultured in RPMI (Gibco 11875) with 10% heat-inactivated FBS and 0.1 mM 2-mercaptoethanol. RAW 264.7 cells were cultured in DMEM (Sigma-Aldrich D5796) with 10% heat-inactivated FBS and 1 mM sodium pyruvate. Each cell line in an exponential growth phase was coincubated either with **sLDL-F** (with apoB100-mimetic peptide) or **LNP-F** (without apoB100-mimetic peptide) particles for 5 h or overnight at 37 °C. After washing with PBS(−), the fluorescence intensity per cell was recorded by KEYENCE Fluorescence Microscope BZ-X700 (KEYENCE Co., Osaka, Japan) using a 470 nm excitation wavelength.

## Results and Discussion

### Preparation of nLDL-Based Imaging Probes

The nLDL-based MRI-CA (**nLDL-Gd**) was prepared according to the previously reported method.^13^ Briefly, **nLDL-Gd** was prepared by initial intercalation of a cyclen-oleic acid derivative (**DO3A-OA**, Figure 1a) into the lipid monolayer of freshly isolated nLDL and subsequent on-surface Gd^3+^ chelation (Figure 1a). After careful removal of free unbound Gd^3+^ by the addition of a tropolone chelator, **nLDL-Gd** with a payload of ca. 200 Gd^3+^ per particle as estimated by ICP-MS was successfully obtained. No aggregation was observed by DLS and cryo-EM analyses upon functionalization, remaining ca. 20 nm in diameter, and *r*_1_ relaxivity of ca. 20 s^–1^ mM^–1^ (per Gd^3+^) was determined. To assess nLDL uptake in the atheroplasma of mouse models *in vivo*, a commercially available nLDL-based fluorescent probe (**nLDL-Dil**) was employed.

### Preparation of sLDL-Based Imaging Probes by Surface KAT Ligation

Details were described previously.^21,22^ Briefly, sLDL-based imaging probes were prepared by surface functionalization of the core platform **LNP-KAT** using KAT ligation (Figure 1b). KAT ligation offers several advantages,^25^ including (1) no involvement of coupling agents and no generation of toxic byproduct, (2) rapid kinetics to form natural amide bonds quantitatively, and (3) excellent efficiency even under physiological conditions, rendering it suitable for reactions involving biomaterials and self-assembled materials (*e.g.*, LNP). Furthermore, KAT ligation operates in a chemoselective manner enabling conjugation reactions of biomolecules (*e.g.*, peptides) having various unprotected functional groups. Our recent study confirmed that KAT ligation works rapidly not only in homogeneous systems but also with heterogeneous substrates, including self-assembled monolayers on gold,^27^ further highlighting its suitability for surface functionalization of the nanoparticles.

For the preparation of **LNP-KAT**, a mixture of three commercially available lipids, phosphatidylcholine (**PC**), triolein (**TO**), and cholesteryl oleate (**CO**), and a synthetic oleyl-KAT derivative (**OA-KAT**), was sonicated and extruded in buffer (Figure 1b, *Step I*). The surface functionalizations of the **LNP-KAT** with HA derivatives were performed in a pH 5.2 buffer to form a stable covalent attachment of biomolecules. Several types of LDL-mimetic imaging probes (**sLDL-Gd**, **sLDL-GdR**, and **sLDL-F**) were prepared from this **LNP-KAT** platform by the reactions with a mixture of HA derivatives (1.2 equiv in total) of apoB100-mimetic peptide (**HA-peptide**, Arg-Leu-Thr-Arg-Lys-Arg-Gly-Leu-Lys) and imaging probe (**HA-GdDO3A**, **HA-rhodamine**, or **HA-fluorescein**) in a 1:9 ratio (mol/mol) to provide the sLDL-based probes in high yields (Figure 1b, *Step II*). Morphology of the sLDL after surface KAT ligation revealed a narrow distribution of diameters by DLS (mean ca. 50 nm), ensuring its suitability for administration into model animals.^22^ The results also show that **LNP-KAT** can be a versatile platform for the preparation of a variety of functional LNPs with covalently attached biomolecules on the surface.

### Accumulation of nLDL in the Aorta of Atherosclerosis Mouse Models and Imaging Ability of Plaques by MRI

**Figure 2:**
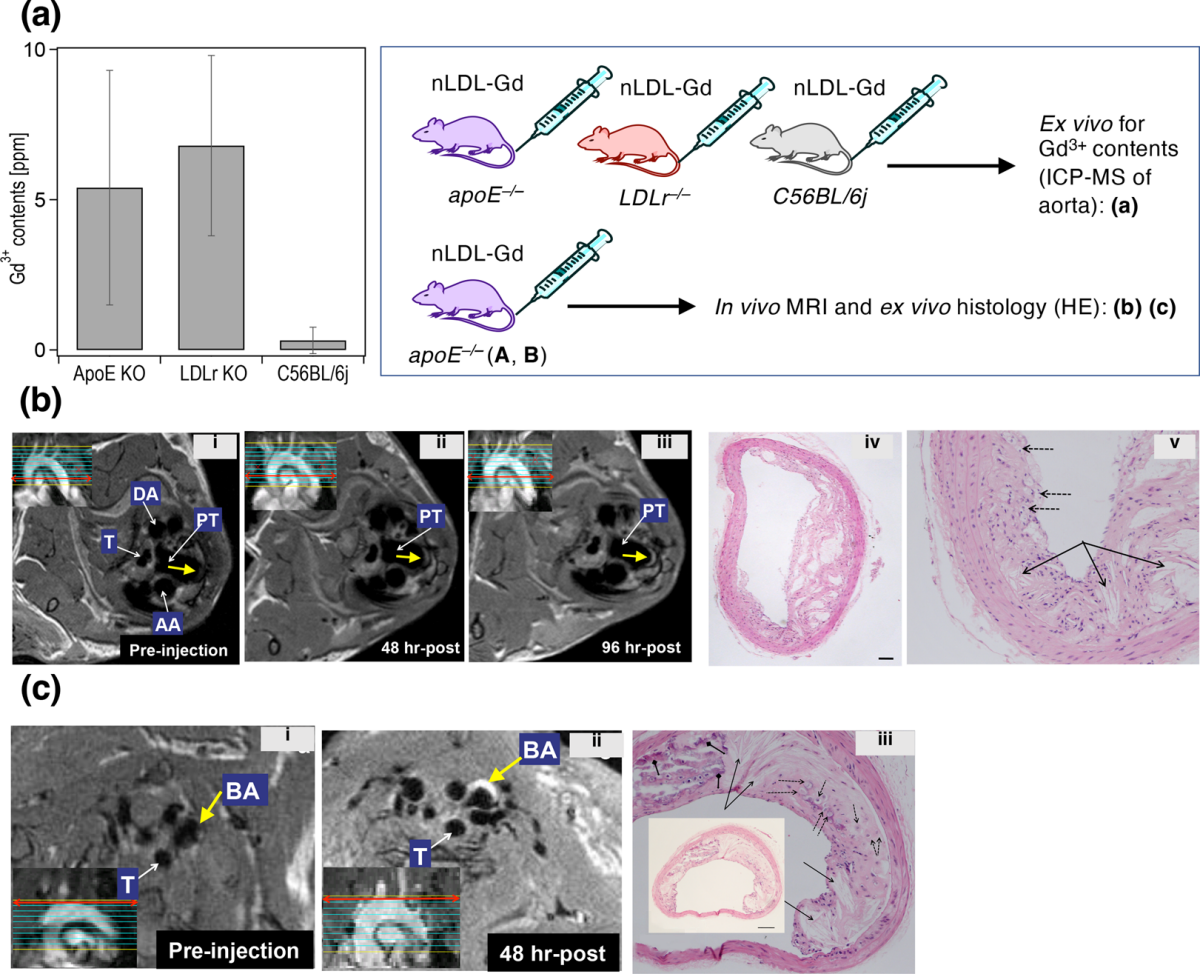
Accumulation of **nLDL-Gd** in atherosclerotic mice aorta and imaging ability by MRI, together with *ex vivo* H&E staining. (a) Gd^3+^ quantification by ICP-MS analyses of dried aorta tissues from *apoE*^*–/–*^, *LDLr*^*–/–*^, or C56BL/6j mice injected with **nLDL-Gd**. Seven samples with dried weights of 0.25 ± 0.14 mg from *apoE*^–/*–*^, 12 samples with dried weights of 0.30 ± 0.14 mg from *LDLr*^–/–^, and 4 samples with dried weights of 0.45 ± 0.26 mg from C56BL/6j were analyzed in the New Bolton Center at the University of Pennsylvania. Error bars represent SE calculated with Excel. (b,c) *In vivo* MRI of two *apoE*^*–/–*^ mice (**A**, **B**) at 0, 48, and 96 h (b-i–iii) after the injection of **nLDL-Gd**, or at 0 and 48 h after the injection of **nLDL-Gd** (c-i, ii), and *ex vivo* histological analysis of the corresponding aorta by H&E staining (b-iv, v, and c-iii). BA: brachiocephalic artery; T: trachea; AA: ascending aorta; DA: descending aorta; PT: pulmonary trunk. Macrophages (dotted arrows), areas containing crystallized cholesterol (arrows), and calcium deposits (arrows with diamond head). For comparison, images in (c-i, ii) are reproduced from our previously published report.^13^ Bars in parts (b-iv) and (c-iii) correspond to 10 μm.

For a preliminary evaluation of the effectiveness of the nLDL vehicle for delivering the probe to the aorta, **nLDL-Gd** was administered to two well-established mouse models for atherosclerosis, *apoE*^*–/–*^ and *LDLr*^*–/–*^, as well as to a healthy mouse model (C56BL/6j) as a control. The *apoE*^*–/–*^ mice present compromised clearance ability for circulating lipids, with consequent accumulation of cholesterol and triglycerides in the bloodstream, resulting in the development of atheroplaques.^28^ Mainly, atherosclerotic lesions in *apoE*^*–/–*^ mice develop along the aorta, in particular, in the aortic root. Similarly, *LDLr*^*–/–*^ mice, which lack a functional LDL receptor, exhibit prolonged circulation time of lipid carriers.^29^ At 48 h postadministration of **nLDL-Gd** to *apoE*^*–/–*^ and *LDLr*^*–/–*^, the accumulation of **nLDL-Gd** in aortic tissue was evaluated by quantifying Gd^3+^ contents by ICP-MS analysis. As shown in Figure 2a, both *apoE*^*–/–*^ and *LDLr*^*–/–*^ accumulated Gd^3+^ with significantly higher concentrations (ca. 5 ppm) compared to the control mice, which presented only a trace amount of Gd^3+^ in their aorta. The results indicated that **nLDL-Gd** particles were accumulated in the diseased aorta of both atherosclerotic mouse models that were expected to possess a sufficient amount of atherosclerotic plaque. In contrast, the same particles were not accumulated in the aorta of healthy mice, which are expected to have a much lower amount of plaques, suggesting that the nLDL promoted the delivery of imaging probes to plaques in animal models to enable therapeutic interventions on the atherosclerotic site.

To evaluate **nLDL-Gd** as an MRI-CA for the imaging of atheroplaques, **nLDL-Gd** particles were administered to two *apoE*^*–/–*^ mice, A and B (Figure 2b,c). At 48 or 96 h postinjection of **nLDL-Gd**, MR images of *apoE*^*–/–*^-**A** revealed significant contrast enhancement in the pulmonary trunk (PT) (Figure 2b-ii, iii) in comparison to the preinjection images (Figure 2b-i), indicating that **nLDL-Gd** facilitated the detection of atheroplaques by MRI. In the other *apoE*^*–/–*^mice (*apoE*^*–/–*^-**B**), a clear enhancement in the brachiocephalic aorta (BA) was observed (Figure 2c-ii), which was not shown in the preinjection image (Figure 2c-i). Importantly, these enhanced MR images were in very good agreements to the *ex vivo* histological data observed in H&E stained sections of the dissected aorta (Figure 2b-iv, c-iii), confirming that contrast enhancement in the MRI directly corresponded to the presence of plaques. In the expanded histological images (Figure 2b-v, c-iii), the presence of macrophages and crystallized cholesterol was observed, suggesting the relation between the enhancement of plaques and the accumulation of **nLDL-Gd** into the macrophages.

**Figure 3:**
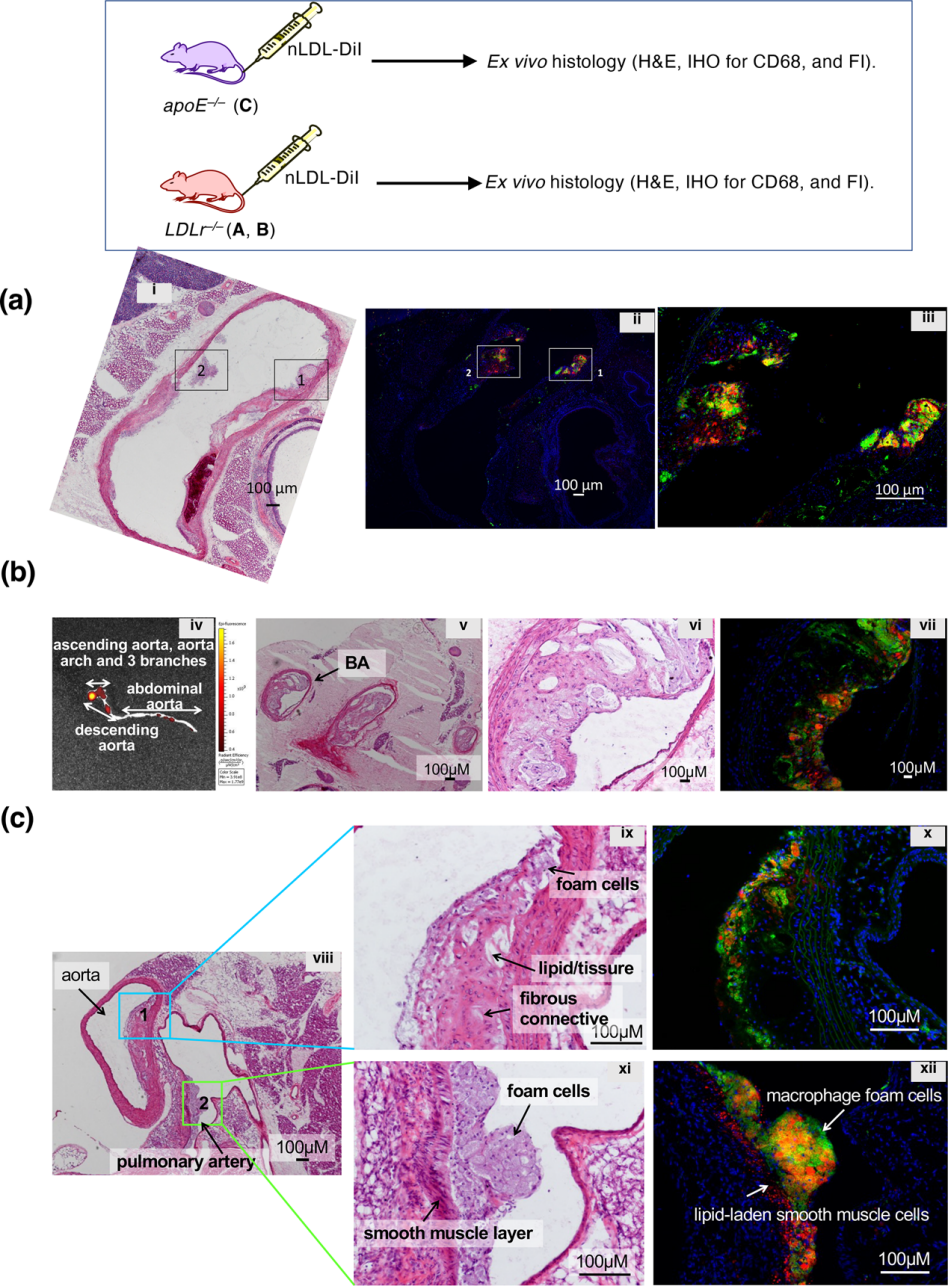
*Ex vivo* histological analyses of aorta sections dissected from (a) one *apoE*^*–/–*^ mouse (*apoE*^*–/–*^-**C**) and (b,c) two *LDLr*^*–/–*^ mice (*LDLr*^*–/–*^-**A** and **B**) injected with **nLDL-Dil** (300 μL, 60 μg, per mouse). The sections (10 μm) were treated by H&E staining or CD60-immunostaining and subjected to optical (i, v, vi, viii, ix, x) or fluorescence (ii, iii, iv, vii, v, vii) microscopies. Fluorescence images for macrophages by CD68 IHC are in green, and **nLDL-DiI** is in red.

To further assess the localizations of nLDL particles in the atherosclerotic plaques, we injected **nLDL-DiI** in both mouse models (*apoE*^*–/–*^-**C** and *LDLr*^*–/–*^-**A** and **B**) and conducted *ex vivo* studies of aortic tissues harvested 72 h after the injection. As indicated by H&E sections of the aorta shown in Figure 3a (*apoE*^*–/–*^-**C**) and Figure 3b,c (*LDLr*^*–/–*^-**A** and **B**), both mouse models presented significant atherosclerosis progression in the aorta, characterized by the presence of foam cells, a lipid core, and a substantial level of calcification in the aortic arch.

In the fluorescence images from the adjacent section to the H&E section, macrophages were stained by CD68 by immunohistochemistry (IHC, in green), while **nLDL-DiI** were in red (Figure 3a-ii, iii, b-vii, c-x, xii). In the magnified view of box-1 and box-2 in Figure 3a-ii, iii, overlapped colocalization of **nLDL-DiI** (red) and macrophages (green) were observed as merged yellow color, confirming the uptake of **nLDL-DiI** by macrophages or macrophage-derived foam cells in *apoE*^*–/–*^. In one *LDL*^*–/–*^ mouse (*LDL*^*–/–*^-**A**), the image of the entire dissected aorta revealed an intense uptake of **nLDL-DiI** in the aortic arch and descending aorta, when compared to the abdominal aorta (Figure 3b-iv). The H&E and immunofluorescence (IF) pictures show discrete colocalization of uptaken **nLDL-DiI** (red) and macrophages (green) in a plaque grown in the brachiocephalic artery (BA) (Figure 3b-vi, vii). Interestingly, the plaque observed in the aorta in other *LDL^–/–^* mouse (**B**) (Figure 3c) appeared as an advanced lesion containing foam cells, lipid crystals, fissures, and fibrous connective tissue (Figure 3c-ix, x), while the plaque in the pulmonary artery was an early-stage lesion containing mainly macrophages/foam cells and lipid-laden smooth muscle cells, which also absorbed **nLDL-DiI** (Figure 3c-xi, xii). Taken together, histological analyses of *in vivo* tests with **nLDL-DiI** showed a preferential accumulation of nLDL in macrophage-rich regions in both *apoE*^*–/–*^ and *LDLr*^*–/–*^ mice. Previous studies have demonstrated the important role of one of the scavenger receptors (SR), SR-A, in the development of atherosclerosis, by assessing the atheroplaque burden on apoE- and SR-A-double-knockout mice.^30^ In these mice, 58% smaller atherosclerotic lesions were observed in comparison to *apoE*^*–/–*^ mice, despite 46% higher level of plasma cholesterol. Similar findings were reported for LDLr- and SR-A-double-knockout mice.^31^ The preferable accumulation of nLDL in this study may presumably occur through SR-mediated uptake. Furthermore, macrophage-rich atheroplaques are often associated with early-stage and vulnerable plaques, highlighting the nLDL’s potential use in identifying and characterizing plaques that are at higher risk of rupture.

### Imaging of Plaques in the Aorta of Atherosclerosis Mouse Models by sLDL-Based MRI-CA

**Figure 4:**
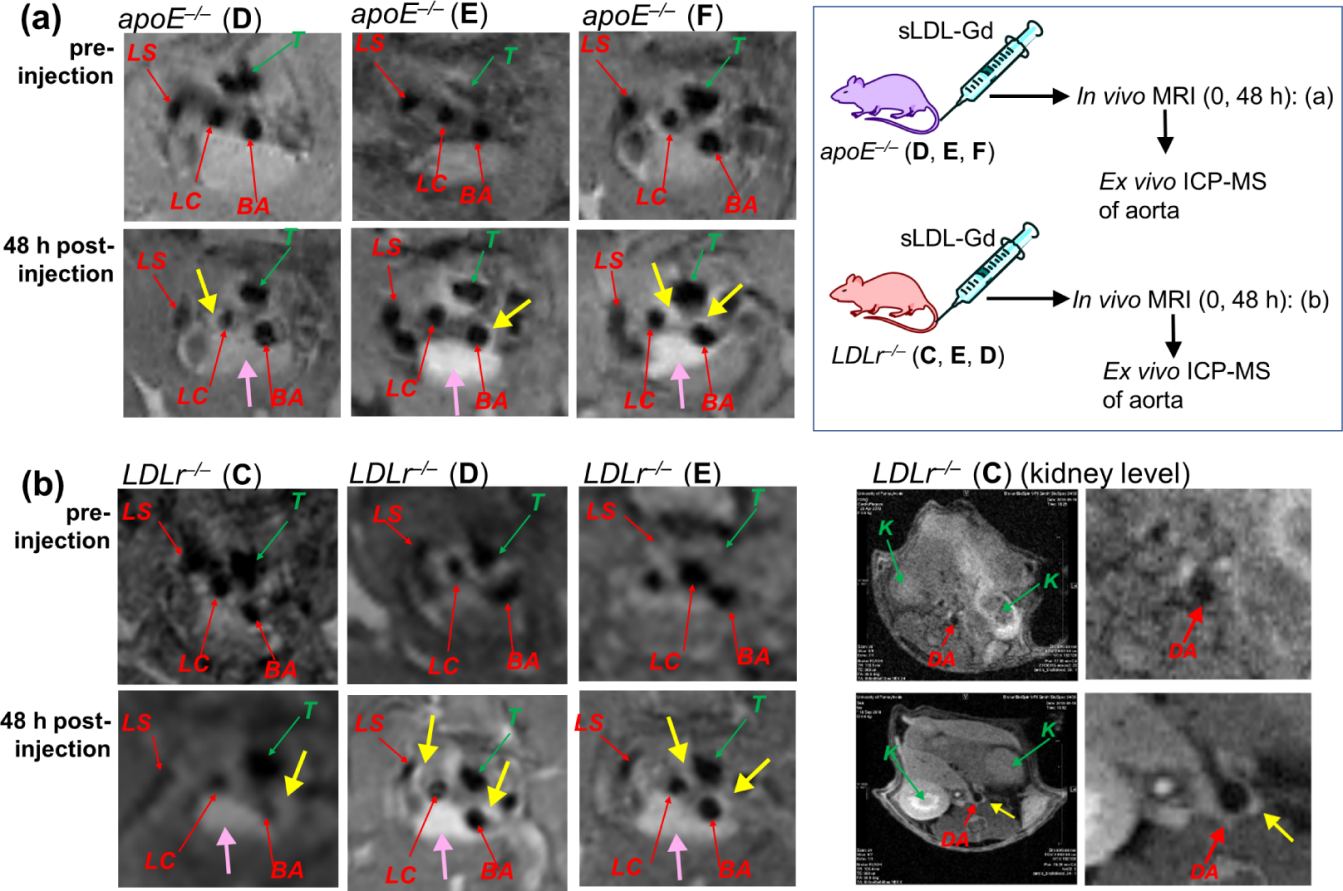
*In vivo* MR images of the aortic arch of atherosclerotic mice *apoE*^*–/–*^ (a) and *LDLr*^*–/–*^ (b) injected with **sLDL-Gd**. MR images of three *apoE*^*–/–*^ mice **D**, **E**, and **F** and three *LDLr*^*–/–*^mice **C**, **D**, and **E** were recorded preinjection and 48 h postinjection of **sLDL-Gd**. The three branches of the aortic arch and the descending aorta are identified by red arrows: BA = brachiocephalic artery; LC = left carotid artery; LS = left subclavian artery; DA = descending aorta. The trachea and kidneys are identified by green arrows: T = trachea; K = kidney. Yellow arrows point to contrast enhancement of the atheroplaque in the BA wall. Pink arrows point to the contrast enhancement of the thymus. Images in (a) are reproduced from the previous study.^22^

By taking into account the above-mentioned ability of nLDL as a delivery system of MRI-CA to atheroplaques resulting in efficient enhancement, we developed an nLDL-mimetic nanoparticle, sLDL, from the combination of commercially available and synthetic lipid components. To mimic the apolipoprotein apoB100 in LDL, a mimetic peptide was covalently attached to the LNP surface. The formed LDL-mimetic LNP was evaluated for its ability to target atherosclerosis. As an initial study, we explored the capability of **sLDL-Gd** in MRI for the visualization of atherosclerotic plaques in three *apoE*^*–/–*^ mice (*apoE*^*–/–*^-**D**, **E**, and **F**) (Figure 4a).^22^ By injecting **sLDL-Gd** with a Gd(III)-chelate dose of 0.018–0.051 mmol·kg^–1^, a distinct contrast enhancement in MR images was observed in the BA and/or left carotid (LC) artery wall of all *apoE*^*–/–*^ mice imaged at 48 h postinjection, similar to the results of **nLDL-Gd** (Figure 2b,c). As the plaques are often observed in these BA and LC artery regions in atherosclerotic model mice, these results indicated that the enhancement was associated with the presence of plaques related to the **sLDL-Gd** preferentially accumulated in plaques *in vivo*. We speculated that the presence of the apoB100-mimetic peptide, containing the sequence of the LDL receptor binding domain, facilitated sLDL accumulation in atheroplaques of *apoE*^*–/–*^ mice by assisting the sLDL uptake through the LDL receptor. In general, it is known that LDL uptake can occur through multiple pathways.^32^ As described above, SRs are known to serve as an alternative pathway that facilitates the accumulation of LDL in macrophages, as also suggested by our experimental data of **nLDL-DiI**, which was effectively accumulated in the plaques of *LDLr*^*–/–*^, presumably *via* the SR pathway (Figure 3).

To have greater insight into the pathway of sLDL accumulation, a more comprehensive evaluation was performed by *in vivo* studies using **sLDL-Gd** on the accumulation in plaques of *LDLr*^*–/–*^ mice. These experiments could provide more information on the sLDL uptake pathways for the accumulation in the atherosclerotic plaques, since sLDL permeation into atheroplaques of *LDLr*^*–/–*^ mice can rely mainly on the SR uptake and/or more general enhanced permeation and retention (EPR) effect of macromolecules.^30,33,34^ We performed MRI experiments on three *LDLr*^*–/–*^ mice (*LDLr*^*–/–*^-**C**, **D**, and **E**) (Figure 4b), following a similar protocol as described for *apoE*^*–/–*^ mice. After the initial MRI baseline acquisition, the **sLDL-Gd** with a Gd(III)-chelate dose of 0.072–0.126 mmol·kg^–1^ was administered, and MR images were acquired again at 48 h postinjection (Figure 4b). The imaging results at the level of the aortic arch obtained for *LDLr*^*–/–*^ mice qualitatively resembled the one observed for *apoE*^*–/–*^ mice, with significant contrast enhancement at the BA wall (Figure 4b). In an image at the kidney level, which was taken in one of the *LDLr*^–/–^ mice (*LDLr*^*–/–*^-**C**), there was a clear enhancement of the descending aorta (DA) (Figure 4b). This result was in line with the data from **nLDL-DiI** particles accumulated in the same region, as shown by the fluorescence image (Figure 3b-iv).

To further obtain more evidence that the contrast enhancement of the plaques observed in the MRI corresponded to the accumulation of **sLDL-Gd** particles, the amount of Gd^3+^ in the aorta was determined by *ex vivo* ICP-MS analyses. After *in vivo* MR imaging, the aorta was harvested and sectioned into two parts for the analyses: (1) the ascending aorta including the entire aortic arch and (2) the descending aorta. Within the same mouse model, the quantities of Gd^3+^ in the aorta were found to correlate with the dosage of **sLDL-Gd** injected (Tables 1 and S1); higher doses of administered **sLDL-Gd** resulted in an increased accumulation of Gd^3+^ in the plaques. When the observed Gd^3+^ contents were evaluated in relation to the **sLDL-Gd** dosage, the accumulated Gd^3+^ contents in all *apoE*^*–/–*^ mice were three times higher in the aortic arch than in the abdominal aorta (Table 1). A similar situation was found in *LDLr*^*–/–*^ mice, with higher accumulation of Gd^3+^ in the aortic arch, in good agreement with the results of **nLDL-DiI** shown in Figure 3b-iv. Interestingly, striking discrepancies were observed when comparing the Gd^3+^ contents in the aorta between the two mouse models, with a much higher Gd^3+^ accumulation observed in the aorta of *LDLr*^*–/–*^ mice compared to that of *apoE*^*–/–*^ mice (Table 1). When the Gd^3+^ contents in the aortic arch and abdominal aorta of *LDLr*^*–/–*^ mice were evaluated relative to the applied dosage, ca. 5–6 times more efficient Gd^3+^ accumulations were observed in *LDLr*^*–/–*^ in comparison to *apoE*^*–/–*^ (Table 1).

**Table 1:** ICP-MS Analysis for Gd Contents in Aorta Tissue from Four *apoE*^*–/–*^ Mice and Three *LDLr*^*–/–*^ Mice at 48 h Postinjection of sLDL-Gd^a^

				Gd contents in aorta					
				aortic arch			abdominal aorta		
mouse strains	dose of Gd [mmol · kg^–1^]	total Gd dose [μmol]	total lipid dose [μmol]	Gd contents by ICP-MS [ppm]	total Gd [nmol]	% delivered	Gd contents by ICP-MS [ppm]	total Gd [nmol]	% delivered
*apoE*^*–/–*^	0.018	0.612	26.1	3.66	0.01862	0.00304	0.98	0.00623	0.00102
	0.051	2.499	106.5	14.6	0.06499	0.00260	5.76	0.02564	0.00103
	0.042	1.596	68.0	4.26	0.02438	0.00153	2.67	0.01358	0.00085
	0.087	3.045	64.9	17.9	0.09107	0.00299	4.12	0.02096	0.00069
*LDLr*^*–/–*^	0.072	2.52	53.7	79.7	0.45615	0.01810	25	0.14308	0.00568
	0.126	4.032	85.9	105	0.80127	0.01987	64.3	0.36801	0.00913

a The injection volumes are ca. 250 μL.

Our findings reveal two important insights into the behavior of sLDL particles in relation to atherosclerotic plaque development and detection. First, sLDL particles can accumulate significantly in atheroplaques regardless of LDLr expression, indicating that alternative mechanisms may facilitate their retention within the vascular lesions. Second, the higher Gd^3+^ accumulation observed for *LDLr*^*–/–*^ mice suggests that sLDL particles preferentially target areas abundant in macrophages. These findings align with the observations for nLDL mentioned above, indicating that sLDLs are valid mimetic analogues of nLDL, retaining the ability to specifically target atheroplaques in both mouse models. Interestingly, the unexpectedly high contrast enhancement observed for the thymus and other macrophage-rich regions suggests that SR-A may play a significant role in regulating sLDL uptake, indicating that sLDL can also be a good vehicle for targeting the vulnerable plaques.^46^

### Biodistribution of sLDL-GdR in *apoE*^*–/–*^ Mice by CryoVIZ Analyses

**Figure 5:**
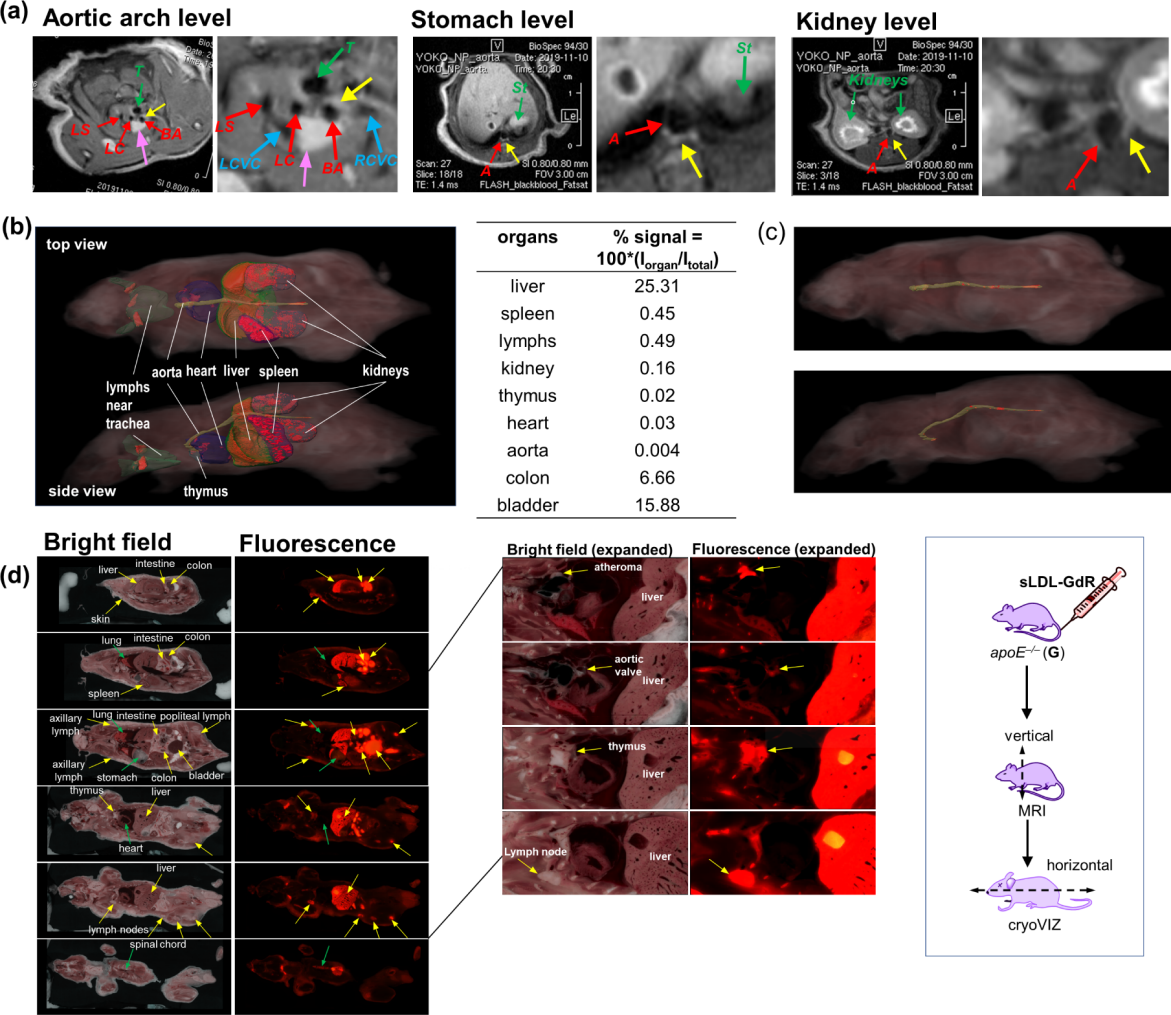
MRI and cryoVIZ imaging of an *apoE*^*–/–*^ mouse (*apoE*^*–/–*^-**G**) injected with **sLDL-GdR**. (a) MRI at 24 h postinjection at the level of aortic arch, stomach, and kidney (left: overall vertical image; right: expansion). Enhanced aorta parts are marked with yellow arrow. (b) Estimated whole-body biodistribution of **sLDL-GdR** and fluorescence intensity ratio in the organs obtained by *ex vivo* cryoVIZ analysis. (c) Fluorescence emission observed along the aorta (upper: top view; lower: side view). (d) Brightfield and fluorescence imaging (left) with a magnified view (right) by *ex vivo* analysis by cryoVIZ.

To evaluate the effectiveness of sLDL as a targeted vehicle to the atherosclerosis plaques and possible clearance pathways, the biodistribution of sLDL was investigated by both *in vivo* MRI analyses and *ex vivo* cryosectioning imaging analyses (cryoViz) on each identical mouse. The **sLDL-GdR** was administered to two *apoE***^–/–^** mice (**G** and **H**). An *apoE*^*–/–*^ mouse (*apoE*^*–/–*^-**G**) was injected with **sLDL-GdR**, with a Gd^3+^ dose of 0.109 mmol·kg^–1^, subjected to MRI (Figure 5a), and then euthanized. Thin-sectioned tissues from dorsal to ventral (coronal view) and the entire body were analyzed by brightfield and fluorescence microscopy (Figure 5b–d). As a result, a considerable accumulation of **sLDL-GdR** in the liver, gastrointestinal tract, kidneys, and bladder was observed (Figures 5b and S21), suggesting that sLDL was cleared out through both hepatic-biliary and renal pathways. Previous studies on the biodistribution of lipid nanostructures in atherosclerotic mice showed preferential localization in liver, while the accumulation in kidneys remained very low, suggesting the hepatic metabolism as the main clearance pathway.^35^ However, it has been shown that fatty acid derivatives can also be metabolized and excreted *via* the renal pathway, which could explain the significant fluorescence signal observed in kidneys and bladder, together with the liver.^36,37^Figure 5c illustrates distinct fluorescent regions lining the aorta, likely to indicate the existence of atheroplaques. This observation suggests the accumulation of fluorescently labeled sLDL in regions associated with atherosclerosis. Interestingly, the full-body cryoViz analysis indicated a strong accumulation of **sLDL-GdR** in several other organs, particularly in regions with a significant presence of macrophages, such as the thymus, lymph nodes, and spleen (Figures 5d and S22).

This observation corroborates our ICP-MS results, further suggesting that the **sLDL-GdR** uptake could be mediated by SR type-A, heavily expressed in macrophages.^38,39^ Importantly, the coronal cryoViz analyses corroborated the MRI findings, showing a clear atheroplaque burden in the regions of the aortic arch and aortic valve in the brightfield images (Figure 5d), to which corresponded a strong fluorescent signal in fluorescence images, indicative of **sLDL-GdR** accumulation (Figure 5d).

**Figure 6:**
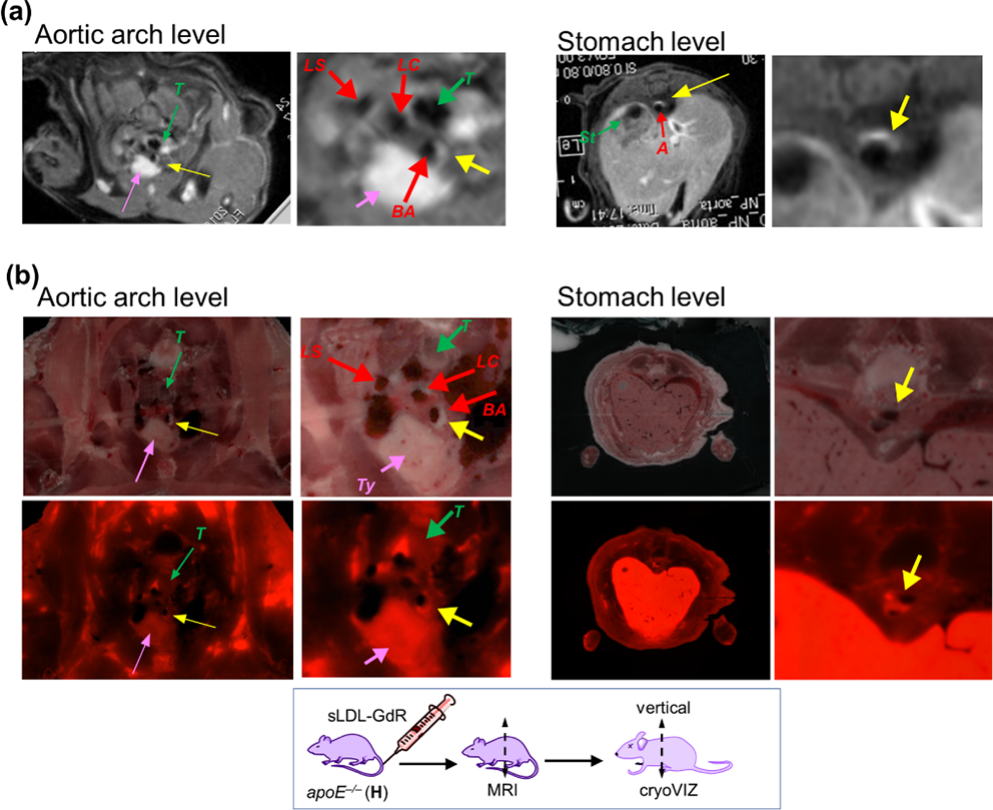
MRI and cryoVIZ imaging of an *apoE*^*–/–*^ mouse (*apoE*^*–/–*^-**H**) injected with **sLDL-GdR**. (a) MRI at 24 h postinjection at the level of aortic arch and stomach (left: overall vertical image; right: expansion). Enhanced aorta parts are marked with yellow arrow. (b) Brightfield and fluorescence imaging (left) with magnified view (right) at the level of the aortic arch and stomach by *ex vivo* analysis by cryoVIZ.

A second *apoE*^*–/–*^ mouse (*apoE*^*–/–*^-**H**) was injected with **sLDL-GdR**, with a Gd dose of 0.087 mmol·kg^–1^. The mouse was subjected to MRI at 24 h postinjection, displaying contrast enhancement of atheroplaques at the level of the aortic arch and the descending aorta (Figure 6a). Subsequently, the mouse was euthanized, and thin sections were obtained from head to tail along the axial plane, orthogonal to the aorta instead of along the coronal plane, as previously done. These sections were examined under both brightfield and fluorescence microscopy to analyze the distribution of **sLDL-GdR** (Figure 6b and Movie S1). Brightfield images showed the presence of atheroplaques occluding the aorta lumen through the entire mouse aorta, to which corresponded a high fluorescence signal, indicating the considerable accumulation of **sLDL-GdR** into the atherosclerotic lesions. In particular, the major atheroplaque burden in the aortic arch was found in the BA, corroborating our MR imaging results. Interestingly, a significant fluorescence signal was also observed in atheroplaques located in the region of the aortic root, an area challenging to image by MRI due to the continuous cardiac motion. The axial sectioning enabled better visualization of the lumen of the descending aorta (Figure 6b and Movie S2). Brightfield images showed the presence of atheroplaques along the entire descending aorta, protruding into the arterial lumen and causing a stenosis, while fluorescence images confirmed the consistent accumulation of **sLDL-GdR** in the imaged atherosclerotic lesions (Figure 6b). Importantly, a remarkable fluorescence signal was observed in the liver, supporting previous findings about the hepatic excretion pathway, and in the thymus and lymph, further underlying the affinity of **sLDL-GdR** for macrophages.

### *In Vitro* Cell Assays of sLDL-F in RAW 264.7 and THP-1

To gain further insight into the mechanism of sLDL accumulation in atheroplaques, we carried out cell culture investigations. An initial key step in atheroplaque formation comprises the adhesion of monocytes on the vascular endothelial damaged site, followed by transmigration into the intima, and differentiation into macrophages that uptake and accumulate lipoproteins, forming foam cells.^2^ sLDL nanoparticles were modified with fluorescein to obtain **sLDL-F**, which were employed for the *in vitro* internalization test in two cell lines, THP-1 monocytes and RAW 264.7 macrophages. Additionally, nanoparticles modified only with fluorescein, without the apoB100 mimetic peptide (**LNP-F**), were prepared in parallel for a control experiment aimed at evaluating the effect of the apoB100-mimetic peptide on the internalization of sLDL.

**Figure 7:**
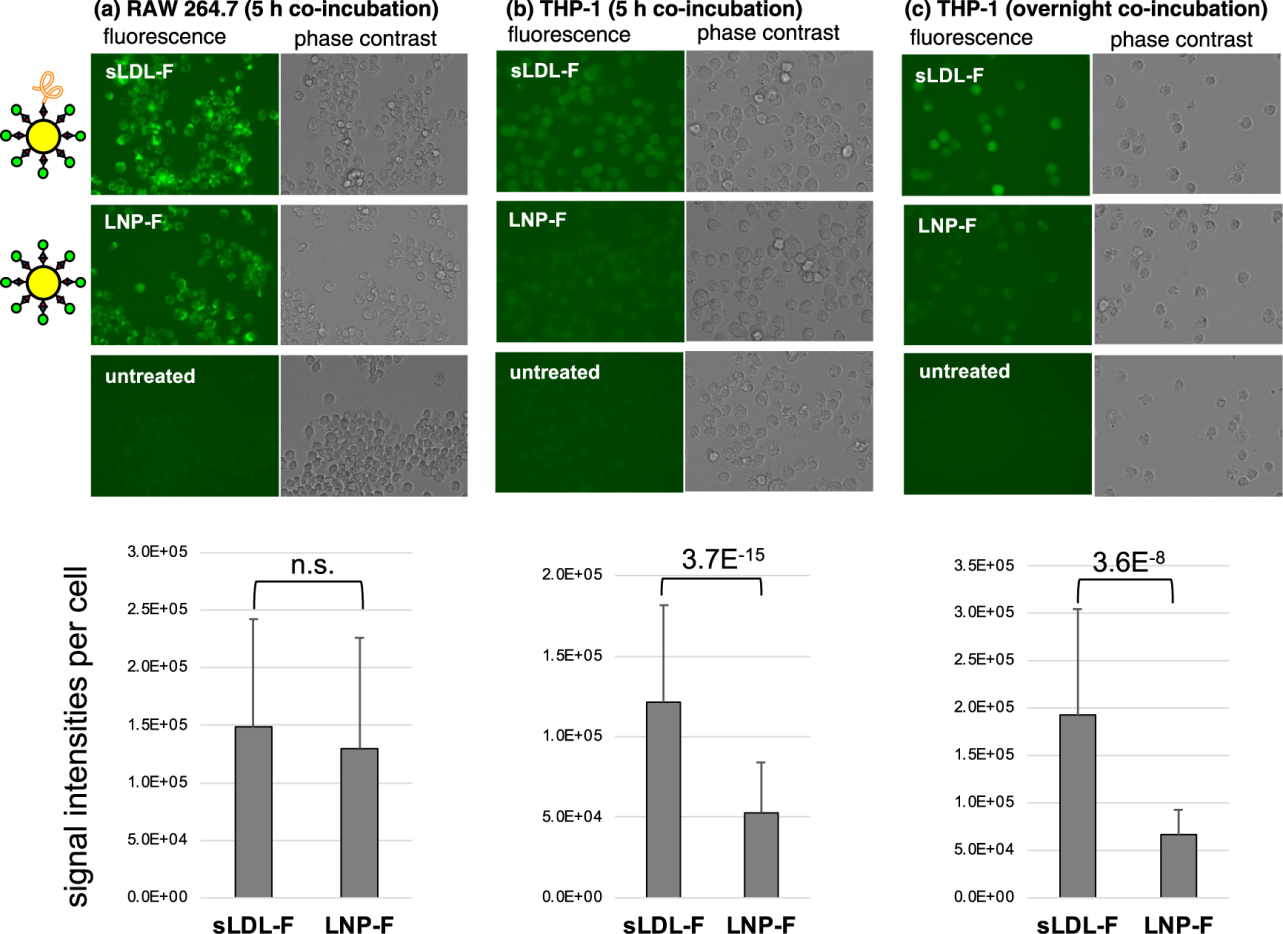
Fluorescence microscopy images for the internalization of **sLDL-F** (with peptide) (top), **LNP-F** (without peptide) (middle), and negative controls (untreated cells, bottom) by RAW 264.7 (a) and THP-1 (b,c) cell lines. Graphs show the quantification of the fluorescence intensities. Each cell line was coincubated with **sLDL-F** or **LNP-F** for 5 h (a,b) or overnight (c), and the incorporation of particles was observed by fluorescence microscopy. Error bars represent standard deviation, and statistical evaluations were performed by unpaired two-tailed Student’s *t*-test (*n* = 95 and 72 (a), 72 and 78 (b), 22 and 35 (c), for **sLDL-F** and **LNP-F**, respectively). The magnification was ×60, including ×3 digital zooming. N.s.: not significant.

As a result, the **sLDL-F** nanoparticles were efficiently internalized in RAW 264.7 cells (Figure 7a, upper row), although the control experiment using **LNP-F** also showed a comparable uptake (Figure 7a, middle row). This result suggests that sLDL internalization into this cell line is not an LDL receptor-mediated type but most likely *via* the scavenger receptor,^30,33,40−42^ or receptor-independent pathways, such as pinocytosis.^39,43^ In contrast, the THP-1 cells showed a more efficient uptake of **sLDL-F** in comparison to **LNP-F**, lacking the apoB100-mimetic peptide (Figure 7b,c), suggesting that the presence of the mimetic peptide can affect particle internalization in some cell types. The internalization of nanoparticles in THP-1 cells may also be regulated by scavenger receptors, which bind to unmodified LDLs (*e.g.*, SR-class B) or pinocytotic engulfment.^44,45^ Recently, Wathiong et al. suggested a novel role of membrane-associated sialic acids in the internalization of nanoparticles in THP-1 cells.^46^ Further studies are required to elucidate the molecular mechanism of the internalization process.

THP-1 cells are among the most used cell lines to investigate the function and regulation of monocytes and macrophages in the cardiovascular system, especially as models for *in vitro* differentiation-driven macrophages.^47^ Their use is attributed to the homogeneous genetic background of THP-1 cells, which minimizes variability in cell phenotype.^42^ RAW 264.7 cells represent a well-established model of macrophages for *in vitro* tests.^43^

The results showed that sLDL was readily internalized by both cell models without inducing significant cytotoxic effects. However, while RAW 264.7 macrophages did not show any preference for sLDL over the control LNP, THP-1 cells showed a clear preference for sLDL, suggesting that the apoB100 mimetic peptide may play a role in mediating the uptake of sLDL in monocytes. Since increased population and reprogramming toward an activated inflammatory response of blood monocytes are observed in *LDLr*^*–/–*^ mice fed with high-fat diet, monocytes may be one of the critical cell types responding to trained immunity associated with hyperlipidemia/hypercholesterolemia and involved in the onset of atherosclerosis. Although the results obtained in this study do not provide definitive evidence on how particles accumulate into the atheroplaques, the uptake of **sLDL-F** by macrophages is in line with that observed in the *in vivo* data by MRI and cryoViz. The uptake of **sLDL-F** by macrophage and monocyte-type cells is consistent with what was observed in the *in vivo* experiments, which showed a considerable sLDL accumulation in macrophage-rich regions such as the thymus and and lymph nodes. Our finding that **sLDL-GdR** is also taken up by lymph nodes and thymus is consistent with the fact that *apoE*^*–/–*^ mice fed a high-fat diet have high levels of inflammation, leading to enrichment of macrophages in atheroplaques and lymphatic tissues. Enhancement in both the aortic wall and surrounding lymphatic sites might be an indication of vulnerable (unstable) plaques, which are prone to rupture.

## Conclusion

In summary, our study explored the use of nLDL and sLDL as vehicles for imaging probes for the *in vivo* MRI detection of atheroplaques using two different atherosclerotic mouse models, *apoE*^*–/–*^ and *LDLr*^*–/–*^. Both nLDL and sLDL proved effective for enhancing the contrast in the MR images of atheroplaques, especially in the BA wall and descending aorta. The *in vivo* MRI results were corroborated by *ex vivo* studies including ICP-MS analyses for Gd content determination, colocalization by immunohistochemistry, and cryoViz imaging for biodistribution. These data revealed that both nLDL and sLDL effectively target the macrophage-rich region of the atheroplaque and also accumulate in other tissues containing macrophages such as the thymus, lymph nodes, spleen, and bone marrow. This is particularly relevant for atherosclerosis detection, where the presence of macrophages can indicate an active inflammation and, therefore, a high-risk atheroplaque formation. The sLDL uptake was further studied *in vitro* in THP-1 monocytes and RAW 264.7 macrophages. Based on the comparable performance of nLDL and sLDL in our data, we will build future work on sLDL as delivery systems for diagnosing and treating inflammatory diseases such as atherosclerosis and lymphomas, which are closely linked with the presence of macrophages.

## Supporting Information Available

Detailed procedures and results for nLDL- and sLDL-based imaging probe preparation, *in vivo* small animal imaging experiments, *ex vivo* 3D cryoVIZ imaging, and *in vitro* cellular experiments (PDF)
Movie of *ex vivo* cryoVIZ imaging 1, axial view, aortic arch (MP4)
Movie of *ex vivo* cryoVIZ imaging 2, axial view, descending aorta (MP4)

## Abbreviations

MRI: magnetic resonance imaging
LDL: low-density lipoprotein
KAT: potassium acyltrifluoroborate
*apoE*^*–/–*^: apoE receptor knockout mouse
*LDLr*^–/–^: LDL receptor knockout mouse
LNP: lipid nanoparticle
GBCA: gadolinium-based contrast agent
HDL: high-density lipoprotein
ICP-MS: inductively coupled plasma mass spectrometry
IHC: immunohistochemistry
DLS: dynamic light scattering
OA: oleic acid
PC: phosphatidylcholine
CO: cholesteryl oleate
TO: triolein
BHT: butylated hydroxytoluene
HA: hydroxylamine
HFD: high-fat diet
LC: left common carotid
LS: left subclavian
BA: brachiocephalic artery
WB: white blood
BB: black blood
ECG: electrocardiogram
FLASH: fast imaging with low angle shot
OCT: optimal cutting solution
RTMI medium: Roswell Park Memorial Institute medium
FBS: fetal bovine serum
DMEM: Dulbecco’s Modified Eagle Medium
H&E: hematoxylin and eosin
PBS: phosphate-buffered saline
PBS(−): phosphate-buffered saline Ca^2+^ and Mg^2+^ free
