# Supplementary material for: Natural and Synthetic LDL-Based Imaging Probes for the Detection of Atherosclerotic Plaques
Source: ACS Pharmacol Transl Sci. 2025 Feb 4;8(2):578–91. doi: 10.1021/acsptsci.4c00667 (PMC11833727; doi:10.1021/acsptsci.4c00667)
Supplement: Supplementary file 1 — pt4c00667_si_001.pdf [file pt4c00667_si_001.pdf]

Natural and Synthetic LDL-Based Imaging Probes for the Detection of Atherosclerotic Plaques

Alessandro Fracassi,[†] Hui Qiao,[‡] Andrew N. Lowell,[∞] Jianbo Cao,[‡] Jeffrey W. Bode,[†] Hisao Masai,[#]* Naoko Yoshizawa-Sugata,[§]* Rong Zhou,[‡]* and Yoko Yamakoshi[†]*

[†]Department of Chemistry and Applied Biosciences, ETH Zürich, Vladimir-Prelog-Weg 3, CH8093 Zürich, Switzerland; [‡]Department of Radiology, Institute for Translational Medicine and Therapeutics, University of Pennsylvania, John Morgan 198, 3620 Hamilton Walk, Philadelphia, PA19104, USA; [∞]Department of Chemistry, Virginia Polytechnic Institute and State University, Davidson Hall, Virginia Tech, 1040 Drillfield Drive, Blacksburg, VA 24061, USA; [#]Department of Basic Medical Sciences, Tokyo Metropolitan Institute of Medical Science, 2-1-6 Kamikitazawa, Setagaya, Tokyo 156-8506, Japan; [§]Research Center for Genome & Medical Sciences, Tokyo Metropolitan Institute of Medical Science, 2-1-6 Kamikitazawa, Setagaya, Tokyo 156-8506, Japan

Corresponding authors: masai-hs@igakuken.or.jp; yoshizawa-nk@igakuken.or.jp; rongzhou@pennmedicine.upenn.edu; yamakoshi@org.chem.ethz.ch

Supporting Information

Table of Contents

1. **Preparation of nLDL and sLDL nanoparticle** 2
   - **1.1 Preparation of nLDL-Gd** 2
   - **1.2 Preparation of lipid nanoparticle with KAT moieties (LNP-KAT)** 3
   - **1.3 Preparation of sLDL-based imaging probes** 4
   - **1.4 Characterization of sLDL particles** 6
2. **Animal models for atherosclerosis apoE$^{-/-}$ and LDLr$^{-/-}$** 7
3. **Ex vivo ICP-MS analyses of Gd^{3+} contents in the aorta of mice injected with sLDL-Gd** 8
4. **In vivo MRI with nLDL-Gd and ex vivo analyses** 8
5. **In vivo MRI and ex vivo analyses of sLDL-based nanoparticles** 9
   - **5.1 In vivo MRI on apoE$^{-/-}$ and LDLr$^{-/-}$ mice with sLDL-Gd and sLDL-GdR** 9
   - **5.2 Ex vivo analysis of the aorta of apoE$^{-/-}$ and LDLr$^{-/-}$ mice by ICP-MS** 18
   - **5.3 Ex vivo analysis by cryoViz brightfield and fluorescence imaging of apoE$^{-/-}$ mice** 18
6. **In vitro cellular incorporation test with sLDL-F and LNP-F** 20

1. Preparation of nLDL and sLDL nanoparticle

1.1 Preparation of nLDL-Gd

The nLDL-based imaging probes were prepared as described below, according to our previous reports.[1,2] The **nLDL**, freshly isolated from the blood of healthy human donors, was treated with a DO3A functionalized with an oleic acid alkyl chain **DO3A-OA** and subsequently with Gd^{3+} (Figure S1). The **nLDL** dispersion (10 mL), isolated in the laboratory of Dr. Lind-Katz in the Children's Hospital of Philadelphia, was filtered through a membrane filter unit (Millex®-GV, 0.22 µm, PVDF, Merck KGaA, Darmstadt, Germany) and dialyzed against 500 mL of pH 7.4 phosphate buffered saline Mg^{2+} & Ca^{2+} free (PBS(–), Thermo Fisher Scientific, Waltham, MA, USA), at 4 °C for 18 h using Spectra/Por4 dialysis tubing with a flat width of 25 mm and a MWCO of 12−14 kD (Spectrum Laboratories, Inc., Rancho Dominguez, CA, USA). The dispersion, recovered from the dialysis tube (11 mL), was placed into a 50 mL conical tube. A solution of **DO3A-OA** (35 mg) in DMSO or in a basic buffer (0.2 mL) was diluted with PBS(–) (0.2 mL) and added to the **nLDL** dispersion above (10 mL, 3.96 mg/mL, 7.42 x 10^{-5} mmol protein, determined by Lowry assay based under the assumption that each nLDL particle contains a single apoB100 protein), and the mixture was incubated at 37 °C for 4 h. The dispersion was filtered again through a membrane filter (Millex®-GV, 0.22 µm, PVDF) and dialyzed against 500 mL of PBS(–) at 4 °C for 18 h using Spectra/Por4 dialysis tubing to remove the unbound **DO3A-OA** providing **nLDL-DO3A** (the **nLDL** with intercalated **DO3A-OA**). A bulk Gd^{3+} citrate solution was prepared by dissolving $GdCl_3 \cdot 6H_2O$ (0.502 g) in concentrated HCl (120 mL) and combining this solution with sodium citrate (2.45 g) in water (480 mL). The resulting solution was acidified to pH 7.4 using aqueous NaOH and aqueous HCl to give a final volume of 650 mL and a concentration of 0.00208 $mmol \cdot mL^{-1}$ of Gd^{3+}. Subsequently, to the dispersion of **nLDL-DO3A** (10 mL,), a solution of Gd^{3+} citrate (50 mL, 0.104 mmol) was added, and the mixture was agitated at 37 °C for 4 h. To remove the unbound free Gd^{3+}, a solution of tropolone (10 mM, 156 mL) in PBS (–) was added and agitated at 4 °C for 4 h. The dispersion was then filtered through a filter unit (Nalgene®, 0.2 µm, PES, 250 mL capacity, Thermo Fisher Scientific) to remove Gd^{3+}-tropolone precipitates, and dialyzed against 1 L of PBS(–) at 4 °C for 16 h to remove excess of free tropolone. The dispersion was filtered through a filter unit (Nalgene®, 0.2 µm, PES, 150 mL capacity), transferred to a centrifugal filter unit (Amicon® Ultra-4, Ultracel-3 Regenerated Cellulose, 3 kDa MWCO, Merk KGaA), to reduce the volume of dispersion by centrifugation with 3000 rpm at 4 °C (Allegra X-15R centrifuge, SX4750 rotor, Beckman Coulter, Inc., Brea, CA, USA) to provide approximately 0.3 mL of **nLDL** functionalized with the Gd(DO3A) derivative (**nLDL-Gd**).

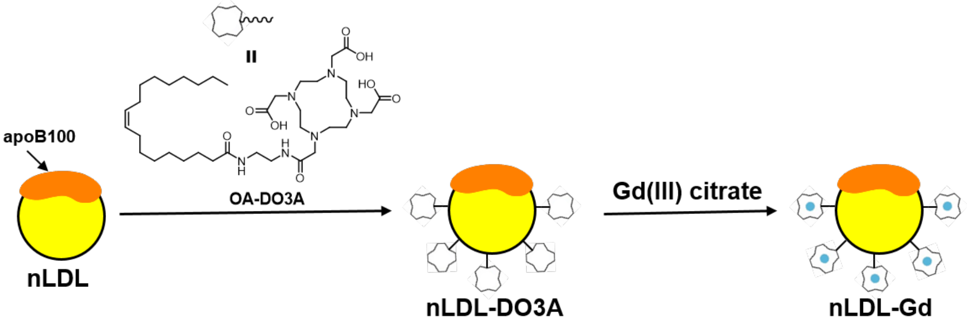

Figure S1. Schematic illustration of **nLDL-Gd** preparation.

1.2 Preparation of lipid nanoparticle with KAT moieties (LNP-KAT)

The lipid nanoparticle with OA-KAT (**LNP-KAT**) was prepared as described below, according to our previous reports.[3,4] To a solution containing a mixture of three lipid substances – phosphatidylcholine from egg yolk (**PC**, 33.8 mg, 45 μmol, Tokyo Chemical Industry, Co. LTD., Tokyo, Japan), triolein (**TO**, 29.2 mg, 33 μmol, Acros Organics, Fischer Scientific Inter. Inc., Pittsburgh, PA, USA) and cholesteryl oleate (**CO**, 9.4 mg, 17 μmol, Alfa Aesar, Fischer Scientific International Inc.), in a ratio of PC-TO-CO (45:33:17, mol/mol/mol) in a mixture of $CHCl_3$-MeOH-acetone (2:1:1, v/v/v) (90 mL), the KAT derivative of oleic acid (**OA-KAT**, either 3.8 mg (10 μmol, 10 mol% of total lipid) or 1.9 mg (5 μmol, 5 mol% of total lipid) in MeOH-acetone (1:1, v/v) (30 mL) was added and thoroughly mixed in a round bottom flask. The solvents were slowly removed by rotary evaporator to make a thin lipid film on the bottom of the flask, which was further lyophilized overnight to remove all trace of organic solvents. To this dried thin film, 4 mL of Tris-HCl buffer (10 mM, pH 8.0, containing KF (10 mM) and BHT (1 mg•L^{-1})) was added, mixed with a vortex mixer for 1 min, and treated under sonication with an ice bath for 2 h using an EMMI® 40HC sonicator (EMAG-AG, Mörfelden-Walldorf, Germany). The obtained suspension of lipid mixture was extruded by a Lipex 10 mL extruder (Lipex Biomembranes, Burnaby, BC, Canada) for 10 times at 50 °C using two stacked polycarbonate membrane filters (pore sizes: 0.05 μm and 0.1 μm). The obtained nanoparticle dispersion was filtered through a filter (0.45 μm, Chromafil® Xtra PTEF-45/25, Macherey Nagel GmbH & Co. KG, Düren, Germany), washed three times with Tris-HCl buffer (10mM, pH 8.0, containing KF (10 mM) and BHT (1 mg•L^{-1})) by a centrifugal filter unit (Amicon® Ultra-0.5, Ultracel-50 Regenerated Cellulose, 50 kDa MWCO) to remove the free unbound lipids, which were not incorporated into the particle. The filtrate **LNP-KAT** particle was kept in Tris-HCl buffer (10 mM, pH 8.0, containing KF (10 mM) and BHT (1 mg•L^{-1})) at 4 ºC before being subjected to the surface functionalization.

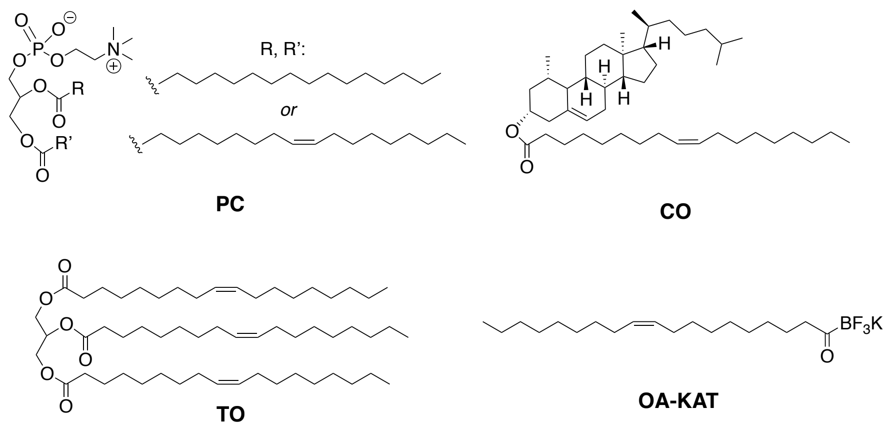

Figure S2. Chemical structures of lipid materials used to prepare the **LNP-KAT** particles.

1.3 Preparation of sLDL-based imaging probes

Preparation of sLDL-Gd. The **sLDL-Gd** was prepared as described below, according to our previously reported procedure (Figure S3).[4] To a mixture of 10 mM KF in phosphate buffer (10 mM, pH 5.8, 32 mL) and 0.1 M HCl (400 μL), a solution of the hydroxylamine-derivative (HA) of the apoB100 mimetic peptide (**HA-peptide**) (0.48 μmol) in H_2O (200 μL) and a solution of the HA derivative of a Gd(DO3A) (**HA-Gd**) (4.32 μmol) in H_2O (700 μL) were added. Subsequently, **LNP-KAT** (5%) particle in 8 mL of Tris-HCl buffer (10 mM, pH 8.0) was added. The pH of the reaction mixture was 5.2. The reaction was stirred overnight at room temperature. The crude mixture was concentrated to a volume of 1.5 mL by spin filtration (Amicon® Ultra-0.5) and washed with 5 mL of PBS(–) (pH 7.4) for three times to remove the unreacted **HA-peptide** and **HA-Gd**. A similar procedure was followed in the preparation of **sLDL-Gd** from **LNP-KAT** (10%). To a mixture of 10 mM KF in phosphate buffer (10 mM, pH 5.8, 36 mL) and 0.1 M HCl (400 μL), a solution of **HA-peptide** (0.48 μmol) in H_2O (200 μL) and a solution of **HA-Gd** (4.32 μmol) in H_2O (700 μL) were added. Subsequently, **LNP-KAT** (10%) particle in 4 mL of Tris-HCl buffer was added. The pH of the reaction mixture was 5.2. The reaction was stirred overnight at room temperature. The crude mixture was concentrated to a volume of 0.5 mL by spin filtration (Amicon® Ultra-0.5) and washed with 2.0 mL of PBS (–) (pH 7.4) for three times to remove the unreacted **HA-peptide** and **HA-Gd**.

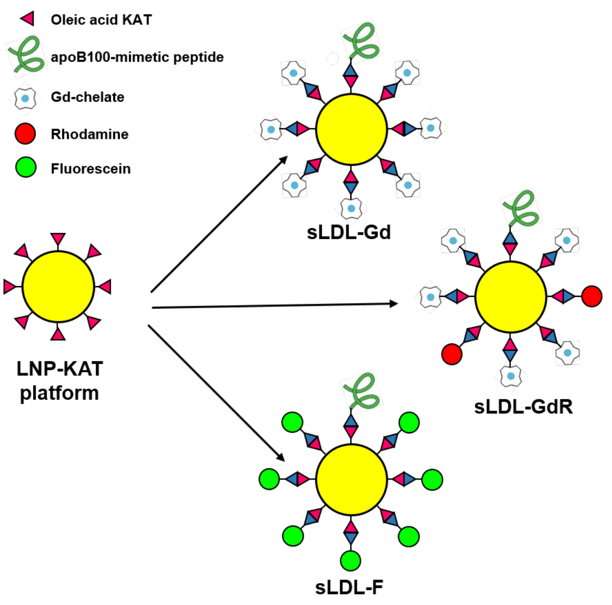

Figure S3. Schematic illustration of the **LNP-KAT** functionalization to obtain sLDL.

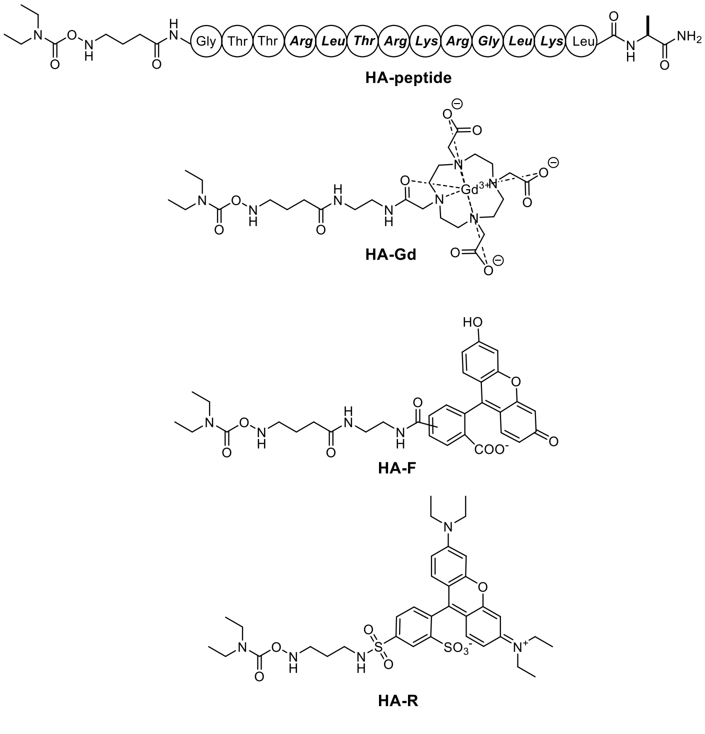

Figure S4. Chemical structures of HA derivatives.

Preparation of sLDL-GdR. The **sLDL-GdR** particle was prepared in a similar manner as **sLDL-Gd**, using **LNP-KAT** (10%) particle as a starting material (Figure S3). To an aqueous solution of a mixture of **HA-peptide** (0.48 µmol), **HA-Gd** (4.08 µmol), and HA derivative of sulforhodamine B (**HA-R**) (0.24 µmol) in a

mole ratio of 10:85:5 in a mixture of pH 5.8 phosphate buffer (10 mM, containing 10 mM of KF) 36 mL and 0.1 M HCl 200 μL, a **LNP-KAT** (10%) dispersion (4 mL in Tris-HCl buffer (10 mM, pH 8.0)) was added. The **sLDL-GdR** was purified as described for **sLDL-Gd** as above.

Preparation of sLDL-F. The **sLDL-F** particle was prepared in a similar manner as **sLDL-Gd**, using **LNP-KAT** (5%) particle as a starting material (Figure S3). To an aqueous solution of a mixture of **HA-peptide** (0.24 μmol), HA derivative of carboxyfluorescein (**HA-F**) (2.16 μmol) in a mixture of pH 5.8 phosphate buffer 16 mL (10 mM, containing 10 mM of KF) and 0.1 M HCl 100 μL, a **LNP-KAT** (5%) dispersion (4 mL in Tris-HCl buffer (10 mM, pH 8.0)) was added. As a control, particle without peptide on the surface (**LNP-F**) was prepared by the reaction of **LNP-KAT** (5%) only with **HA-F** (2.4 μmol). The purifications of **sLDL-F** and **LNP-F** were performed as described for **sLDL-Gd** as above.

1.4 Characterization of sLDL particles

ICP-OES for the determination of Gd contents in sLDL-Gd and sLDL-GdR particles. The ICP-OES analyses of the Gd^{3+} contents were carried out on **sLDL-Gd** and **sLDL-GdR** particles to determine the Gd amounts. Samples were analyzed on a Horiba Ultra 2 inductively coupled plasma-optical emission spectrometer (Horiba Ltd., Kyoto, Japan). An aliquot (20 μL) of each particle dispersion was dissolved in 1 mL of 2% HNO_3 (v/v) (0.3 M) before subjecting to ICP-OES analyses. Calibration curves were prepared using standard solutions Gd (Gd ICP standard (Thermo Fisher (Kandel) GmbH, Kandel, Germany) in a concentration range of 5-200 μM.

DLS analyses. DLS measurements were performed on a Malvern Nano-ZetaSizer (Malvern Instruments Ltd., Worcestershire, UK), equipped with a 5 mW HeNe laser (wavelength: 632.8 nm) and a digital logarithmic correlator. The normalized intensity autocorrelation functions were measured at an angle of 173°. An aliquot (20 μL) of each obtained particle was diluted with pH 8.0Tris-HCl buffer (10 mM,) or pH 7.4 PBS(–) to 0.8 – 1 mL for the measurements, which were carried out at 25 ºC.

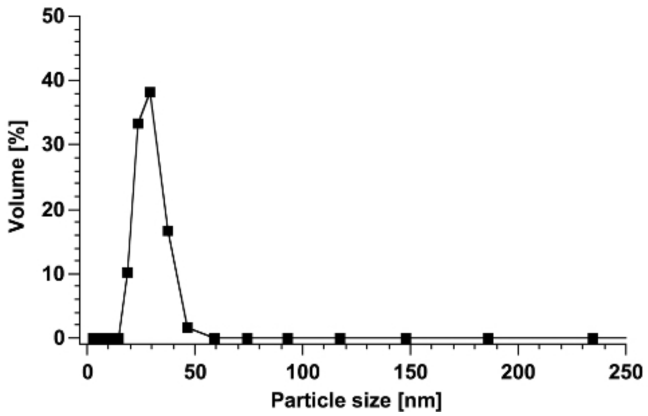

Figure S5. DLS of **nLDL-Gd** in pH 7.4 PBS(–).

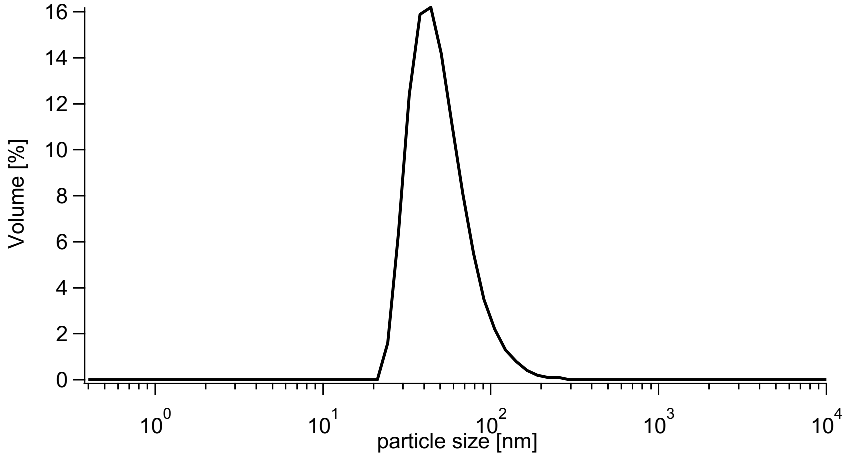

Figure S6. DLS of **sLDL-Gd** in pH 7.4 PBS(–).

2. Animal models for atherosclerosis *apoE*$^{-/-}$ and *LDLr*$^{-/-}$

Two types of atherosclerotic mouse models, *apoE*$^{-/-}$ and *LDLr*$^{-/-}$ were used for the *in vivo* and *ex vivo* studies below. All the animal procedures were approved by the Institutional Animal Care and Use Committee (IACUC) of the University of Pennsylvania. The male *apoE*$^{-/-}$ and *LDLr*$^{-/-}$ mice were purchased from the Jackson Laboratory. The mice started receiving high fat diet (HFD) (42% kcal from fat, Teklad#88137) at 8 weeks of age and the HFD was continued for two months.

3. *Ex vivo* ICP-MS analyses of Gd^{3+} contents in the aorta of mice injected with sLDL-Gd

Table S1. ICP-MS analysis of aorta tissue 48 h post-injection of **sLDL-Gd**.

mouse strains	mouse body weight [g]	dose of Gd [mmol / kg]	total Gd dose [μmol]	total lipid dose [μmol]	Gd contents in aorta							
					a*				b*			
					dry weight of tissue used [mg]	Gd contents by ICP-MS [ppm]	total Gd [nmol]	% delivered	dry weight of tissue used [mg]	Gd contents by ICP-MS [ppm]	total Gd [nmol]	% delivered
apoE$^{-/-}$	34	0.018	0.612	26.1	0.8	3.66	0.01862	0.00304	1.0	0.98	0.00623	0.00102
	49	0.051	2.499	106.5	0.7	14.6	0.06499	0.00260	0.7	5.76	0.02564	0.00103
	38	0.042	1.596	68.0	0.9	4.26	0.02438	0.00153	0.8	2.67	0.01358	0.00085
	35	0.087	3.045	64.9	0.8	17.9	0.09107	0.00299	0.8	4.12	0.02096	0.00069
LDLr$^{-/-}$	35	0.072	2.52	53.7	0.9	79.7	0.45615	0.01810	0.9	25	0.14308	0.00568
	32	0.126	4.032	85.9	1.2	105	0.80127	0.01987	0.9	64.3	0.36801	0.00913

*a: aortic arch, b: abdominal aorta.

4. *In vivo* MRI with nLDL-Gd and *ex vivo* analyses

The mice were injected with **nLDL-Gd** nanoparticle dispersion and subjected to MR imaging. All the procedures were carried out in accordance with the *in vivo* animal protocols approved by the Institutional Animal Care and Use Committee at the University of Pennsylvania. Prior to the injection, the **nLDL-Gd** sample was concentrated in filter tubes (Aimcon Ultra-4) by centrifugation (3000 rpm at 4 °C) to a suitable volume (≤0.5 mL) for tail vein injection into a mouse. No aggregation was detected after the centrifugation. Animals were positioned prone in a quadrature volume 1H coil (i.d. = 3.5 cm, length = 8 cm), which was interfaced to a 9.4 T horizontal bore MR spectrometer (DirectDrive®, Varian, Palo Alto, CA). During imaging, the animal was sedated with 0.8−1% of isoflurane, and its vital signs (including ECG, respiration, and core temperature) were monitored. The rectal temperature was maintained at 36 °C by directing warm air into the magnet bore. “White Blood” (WB) images, where the blood signal in the aorta lumen is not suppressed, were acquired using a multislice gradient echo sequence (TR = 1 heart beat, about 120 ms, TE = 2.4 ms), while “Black Blood” (BB) images, where the blood signal is suppressed, were obtained using a multislice fast spin echo sequence (TR = 1000 ms, TE =10.4 ms, echo train length =1). For both WB and BB images, field of view (FOV) = 26 Å~ 26 mm2, matrix size = 256 Å~ 192, and slice thickness = 0.5 mm. The percent of normalized signal enhancement, %NSE,29,30 was measured for enhanced versus unenhanced artery walls on MR images. After imaging, animals were euthanized and subjected to the histological analyses.

5. In *vivo* MRI and *ex vivo* analyses of sLDL-based nanoparticles

5.1 *In vivo* MRI on *apoE*$^{-/-}$ and *LDLr*$^{-/-}$ mice with sLDL-Gd and sLDL-GdR

Atherosclerotic mouse models and *in vivo* injection of sLDL-Gd or sLDL-GdR. Prior to injection, the **sLDL-Gd** or **sLDL-GdR** samples were concentrated in filter tubes (50 kDa cutoff) by centrifugation (1000 rcf at 4 °C) to a suitable volume (0.25 mL) for the injection to a mouse. No aggregation was observed after the centrifugation. Samples were injected under anesthesia, *via* a catheter placed in the tail vein of the mouse.

***In vivo* MRI tests.** All MRI was performed on a 9.4 T horizontal bore MR spectrometer (DirectDrive®) equipped with a 12-cm (ID) gradient coil. The mouse was positioned prone in a quadrature volume radio frequency (RF) coil (ID = 3.5 cm, length = 8 cm, m2m imaging/Polarean) tuned to ^{1}H resonance frequency (400 MHz). During imaging, the mouse was sedated with 0.8−1% of isoflurane mixed with air (flow rate = 1 L/min). ECG, respiration and core temperature of the mouse were monitored by an MRI-compatible vital sign monitoring system (SA Inc, Long Island, New York). The rectal temperature was maintained at 37 ±0.2 °C by a feedback loop that turns on/off warm air directed into the magnet bore. Scout images were acquired to capture the aortic arch with three rising branches (left carotid (LC), left subclavian (LS), and brachiocephalic artery (BA)). Then the image plane was placed to cut through the three aortic branching points. This image plane was used to acquire the "White Blood" (WB), where the blood signal in the lumen of aorta is not suppressed, as well as "Black Blood" (BB) images, where the blood signal is suppressed (thus black). To obtain WB image, ECG-gated multi-slice gradient echo sequence (TR = 1 heartbeat, about 120 ms, TE = 2.4 ms) was employed with FOV = 26 x 25 mm, matrix size = 192 x 128, slice thickness = 0.8 mm. To obtain BB images, ECG-gated multi-slice FLASH (Fast imaging with Low Angle Shot) (TR = 76 ms, TE =0.97 ms, FA=40 degree) was applied to the same slice. Due to the long interval between MRI sessions, we relied on the unique anatomy of the aortic arch, its branching points, and other thoracic arteries in the imaging plane for the comparison of pre- and post-injection images. For images acquired after **sLDL-Gd or sLDL-GdR** injection, BB was further combined with fat suppression option in the MRI protocol although such option is not necessary to detect enhancement.

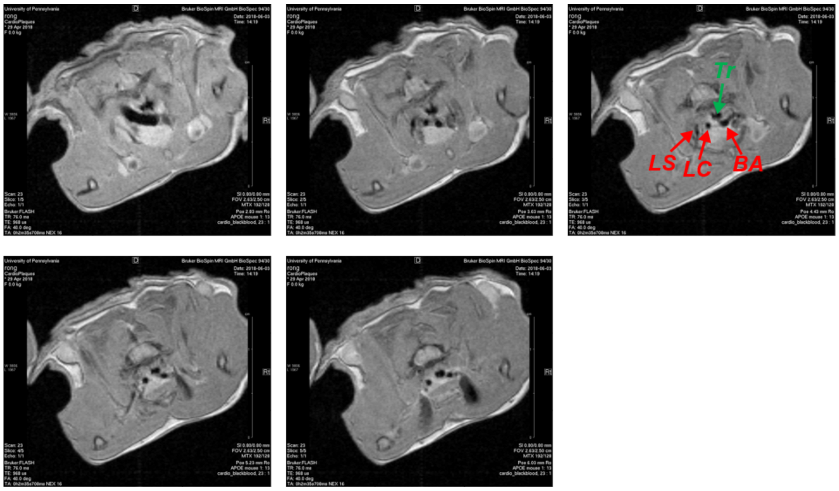

Figure S5. *In vivo* MR images of the aortic arch of an *apoE*$^{-/-}$ mouse (*apoE*$^{-/-}$-**D**), before **sLDL-Gd** injection. The three branches of the aortic arch and the aorta were identified by red arrows: BA = brachiocephalic artery; LC = left carotid artery; LS = left subclavian artery. The trachea is identified by a green arrow.

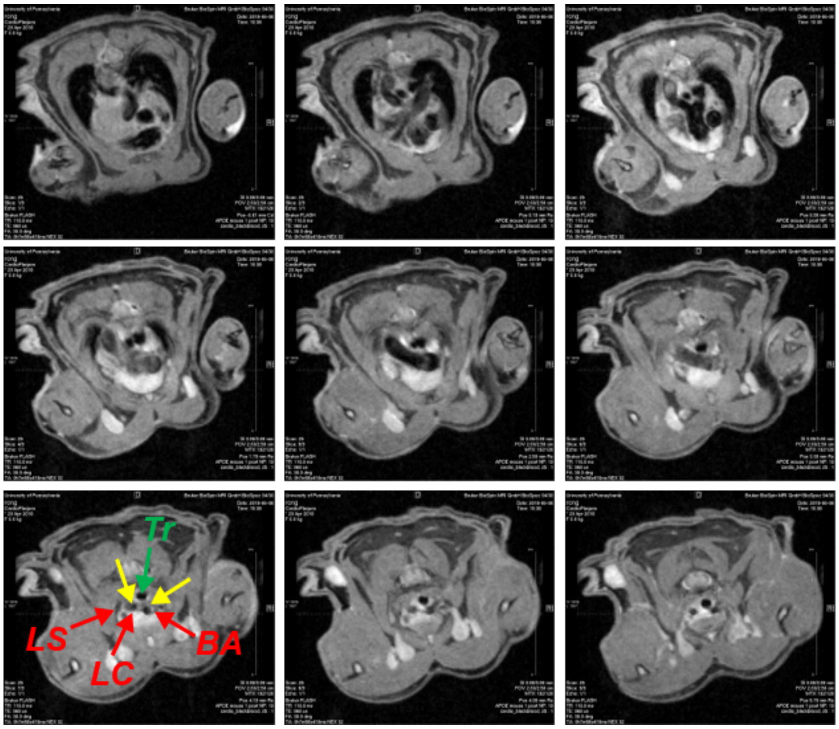

Figure S6. *In vivo* MR images of the aortic arch of the *apoE*$^{-/-}$ mouse (*apoE*$^{-/-}$-**D**), at 48 h after **sLDL-Gd** injection. The three branches of the aortic arch and the aorta were identified by red arrows: BA = brachiocephalic artery; LC = left carotid artery; LS = left subclavian artery. The trachea is identified by a green arrow. Yellow arrows point to contrast enhancement of atheroplaques.

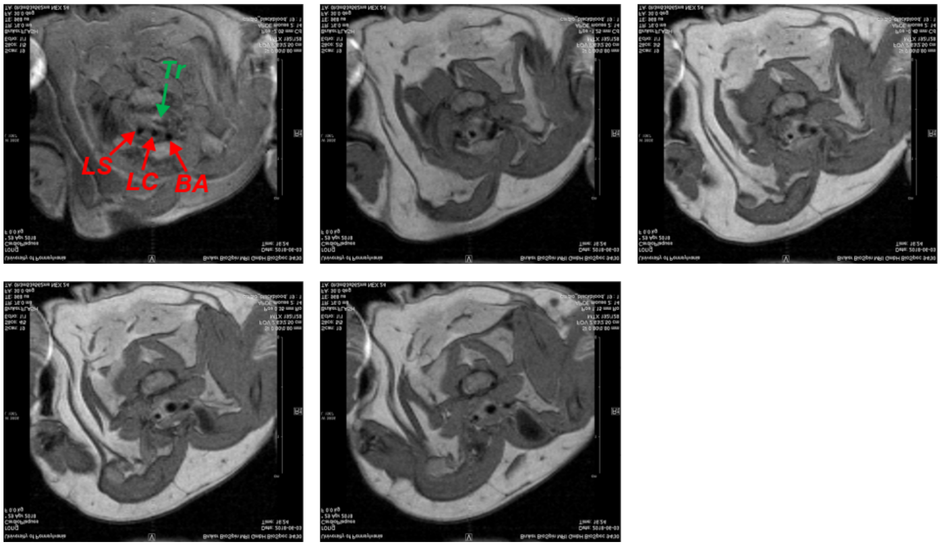

Figure S7. *In vivo* MR images of the aortic arch of an *apoE*$^{-/-}$ mouse (*apoE*$^{-/-}$**-E**), before **sLDL-Gd** injection. The three branches of the aortic arch and the aorta were identified by red arrows: BA = brachiocephalic artery; LC = left carotid artery; LS = left subclavian artery. The trachea is identified by a green arrow.

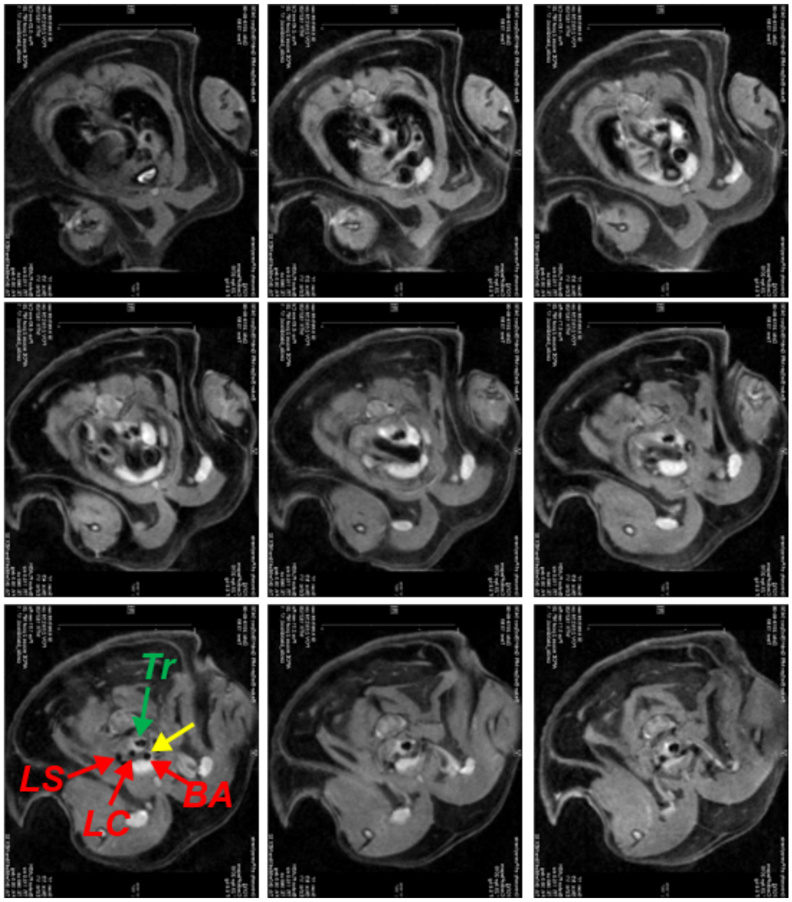

Figure S8. *In vivo* MR images of the aortic arch of the same *apoE*$^{-/-}$ mouse (*apoE*$^{-/-}$**-E**), at 48 h after **sLDL-Gd** injection. The three branches of the aortic arch and the aorta were identified by red arrows: BA = brachiocephalic artery; LC = left carotid artery; LS = left subclavian artery. The trachea is identified by a green arrow. Yellow arrows point to contrast enhancement of atheroplaques.

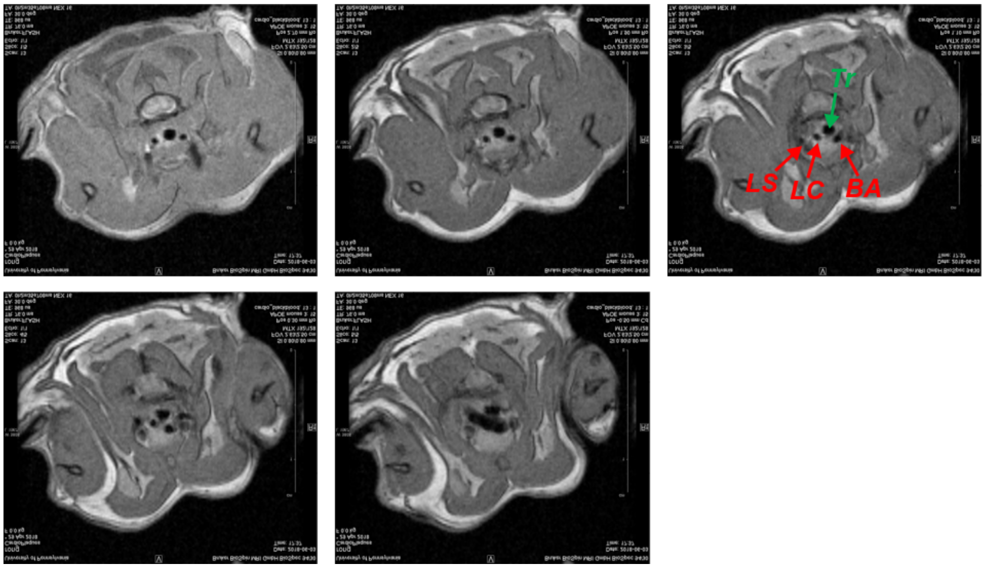

Figure S9. *In vivo* MR images of the aortic arch of an *apoE*$^{-/-}$ mouse (*apoE*$^{-/-}$**-F**), before **sLDL-Gd** injection. The three branches of the aortic arch and the aorta were identified by red arrows: BA = brachiocephalic artery; LC = left carotid artery; LS = left subclavian artery. The trachea is identified by a green arrow.

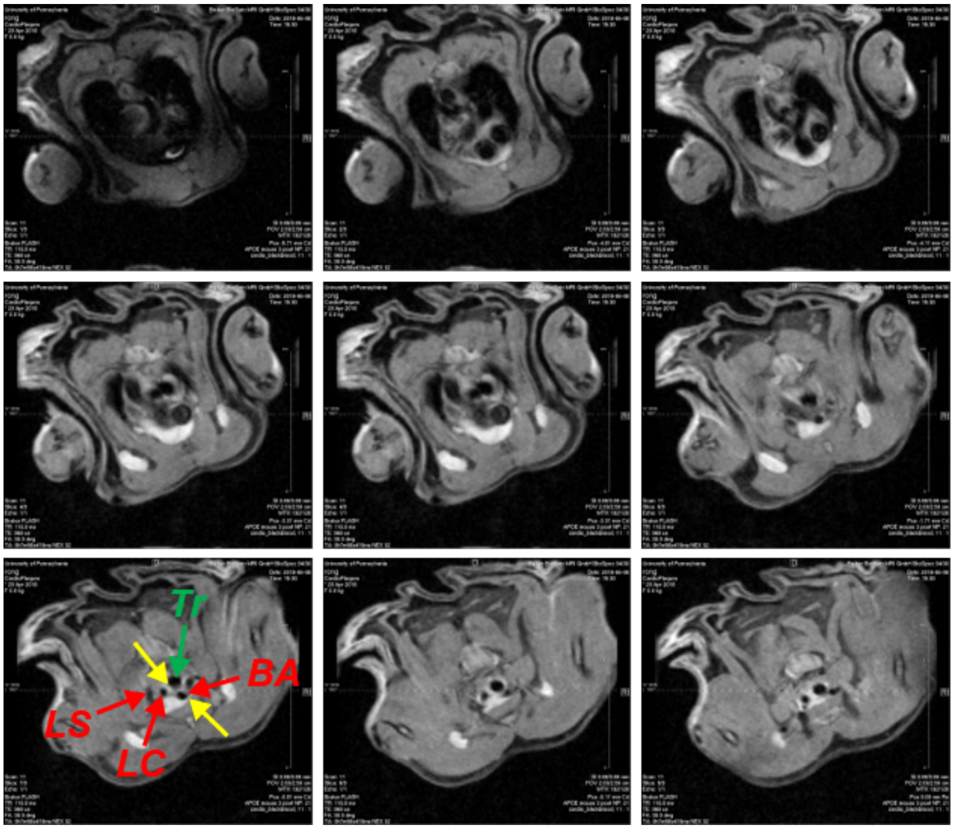

Figure S10. *In vivo* MR images of the aortic arch of the same *apoE*$^{-/-}$ mouse (*apoE*$^{-/-}$**-F**), at 48 h after **sLDL-Gd** injection. The three branches of the aortic arch and the aorta were identified by red arrows: BA = brachiocephalic artery; LC = left carotid artery; LS = left subclavian artery. The trachea is identified by a green arrow. Yellow arrows point to contrast enhancement of atheroplaques.

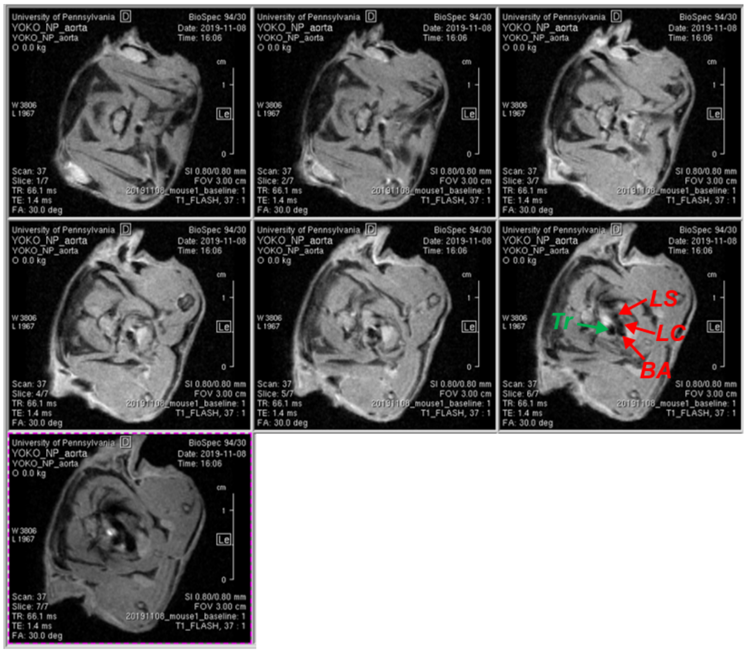

Figure S11. *In vivo* MR images of the aortic arch of an *apoE*$^{-/-}$ mouse (*apoE*$^{-/-}$**-G**), before **sLDL-Gd** injection. The three branches of the aortic arch and the aorta were identified by red arrows: BA = brachiocephalic artery; LC = left carotid artery; LS = left subclavian artery. The trachea is identified by a green arrow.

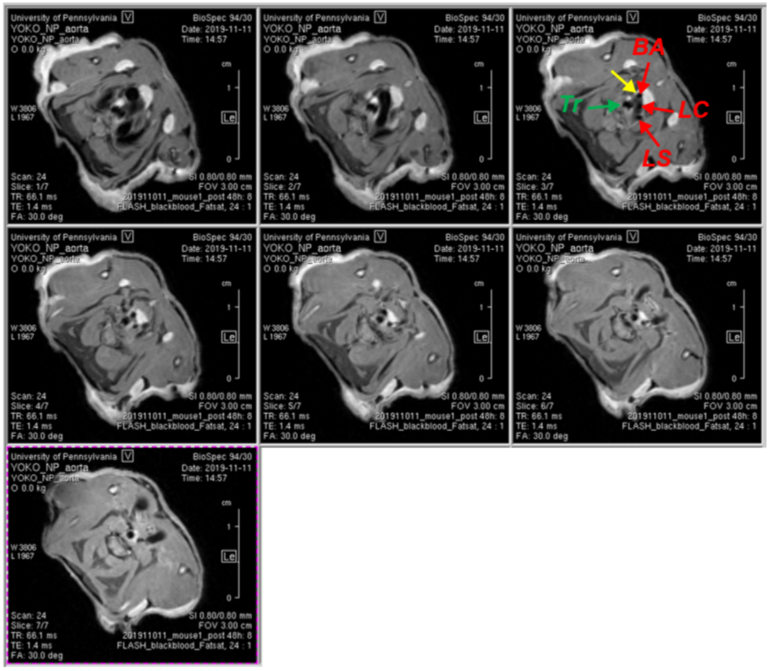

Figure S12. *In vivo* MR images of the aortic arch of the same *apoE*$^{-/-}$ mouse (*apoE*$^{-/-}$**-G**) shown in Figure S11, after **sLDL-Gd** injection. The three branches of the aortic arch and the aorta were identified by red arrows: BA = brachiocephalic artery; LC = left carotid artery; LS = left subclavian artery. The trachea is identified by a green arrow. Yellow arrows point to contrast enhancement of atheroplaques.

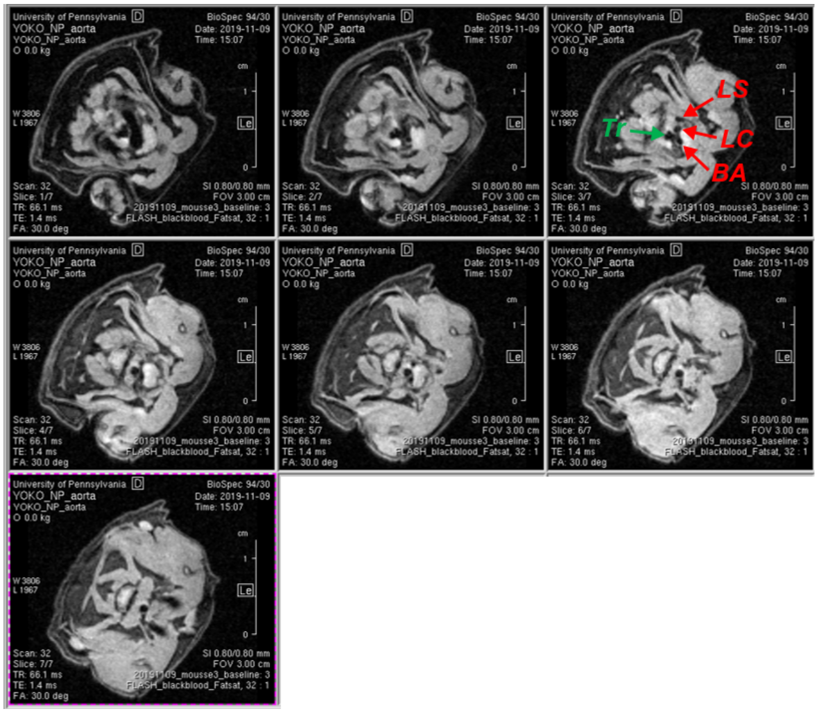

Figure S13. *In vivo* MR images of the aortic arch of an *apoE*$^{-/-}$ mouse (*apoE*$^{-/-}$-**H**), before **sLDL-Gd** injection. The three branches of the aortic arch and the aorta were identified by red arrows: BA = brachiocephalic artery; LC = left carotid artery; LS = left subclavian artery. The trachea is identified by a green arrow.

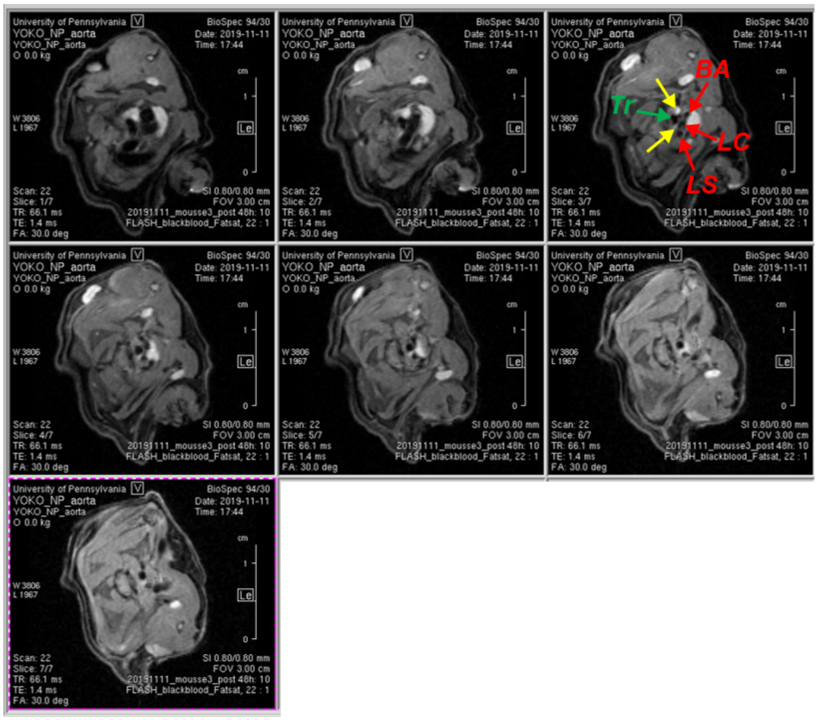

Figure S14. *In vivo* MR images of the aortic arch of the same *apoE*$^{-/-}$ mouse (*apoE*$^{-/-}$-**H**), at 48 h after **sLDL-Gd** injection. The three branches of the aortic arch and the aorta were identified by red arrows: BA = brachiocephalic artery; LC = left carotid artery; LS = left subclavian artery. The trachea is identified by a green arrow. Yellow arrows point to contrast enhancement of atheroplaques.

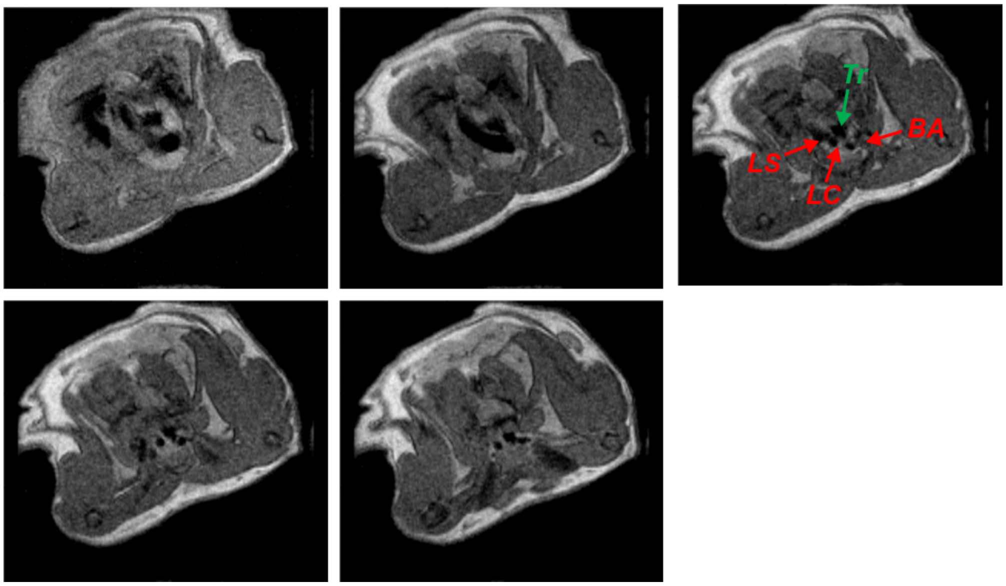

Figure S15. *In vivo* MR images of the aortic arch of an $LDLr^{-/-}$ mouse ($LDLr^{-/-}$**-C**), before **sLDL-Gd** injection. The three branches of the aortic arch and the aorta were identified by red arrows: BA = brachiocephalic artery; LC = left carotid artery; LS = left subclavian artery. The trachea is identified by green arrows. Yellow arrows point to contrast enhancement of atheroplaque in the BA wall.

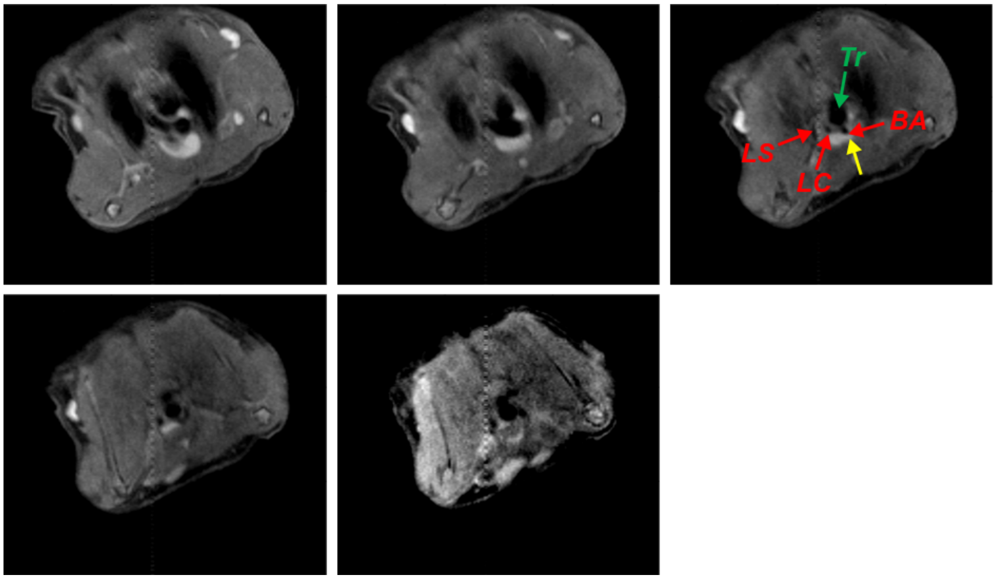

Figure S16. *In vivo* MR images of the aortic arch of the same $LDLr^{-/-}$ mouse ($LDLr^{-/-}$**-C**), at 48 h after **sLDL-Gd** injection. The three branches of the aortic arch and the aorta were identified by red arrows: BA = brachiocephalic artery; LC = left carotid artery; LS = left subclavian artery. The trachea is identified by a green arrow. Yellow arrows point to contrast enhancement of atheroplaques.

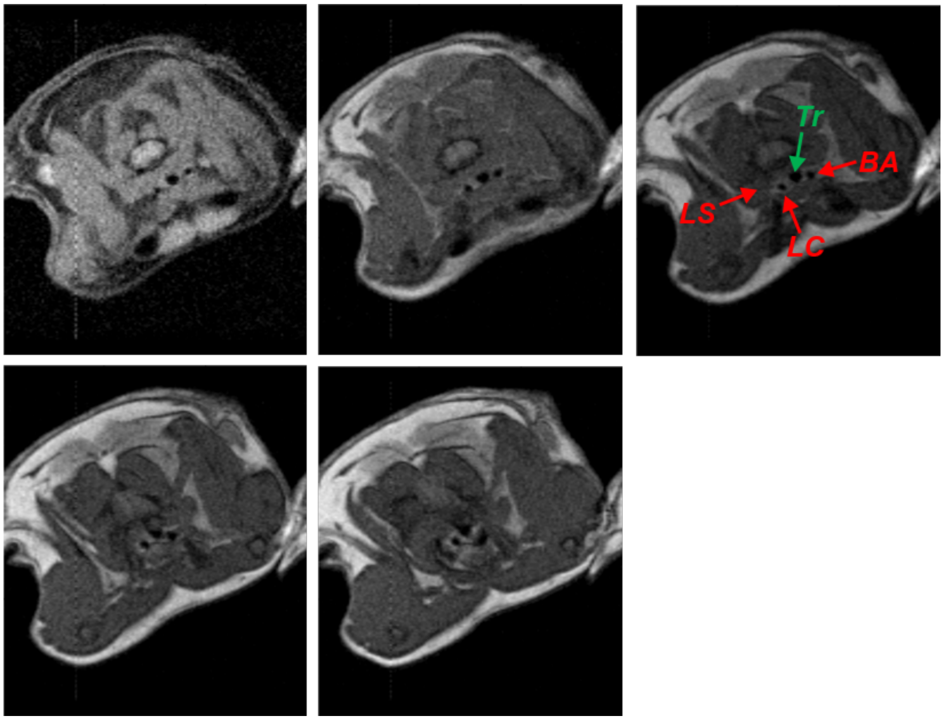

Figure S17. *In vivo* MR images of the aortic arch of an *LDLr*$^{-/-}$ mouse (*LDLr*$^{-/-}$-**D**), before **sLDL-Gd** injection. The three branches of the aortic arch and the aorta were identified by red arrows: BA = brachiocephalic artery; LC = left carotid artery; LS = left subclavian artery. The trachea is identified by green arrows. Yellow arrows point to contrast enhancement of atheroplaque in the BA wall.

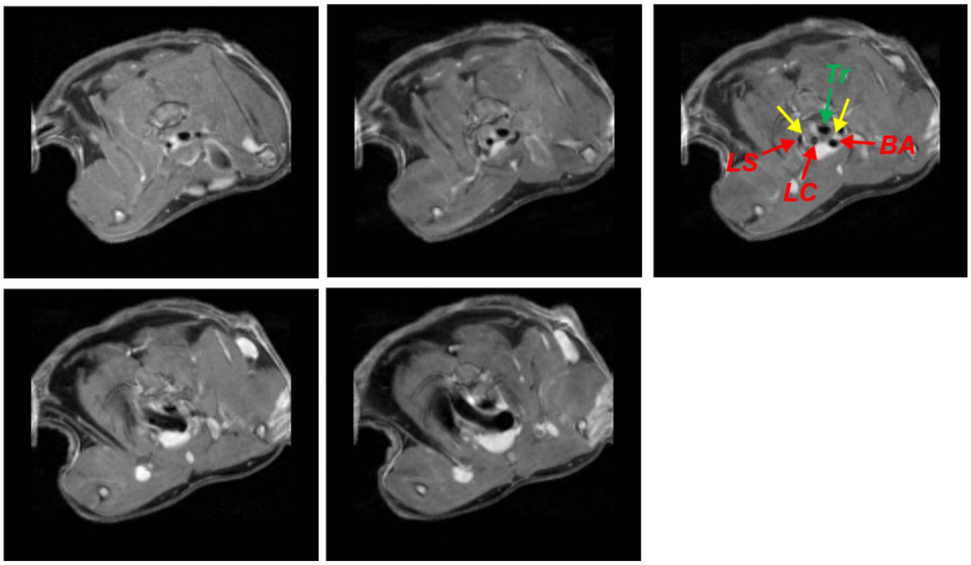

Figure S18. *In vivo* MR images of the aortic arch of the same *LDLr*$^{-/-}$ mouse (*LDLr*$^{-/-}$-**D**), at 48 h after **sLDL-Gd** injection. The three branches of the aortic arch and the aorta were identified by red arrows: BA = brachiocephalic artery; LC = left carotid artery; LS = left subclavian artery. The trachea is identified by a green arrow. Yellow arrows point to contrast enhancement of atheroplaques.

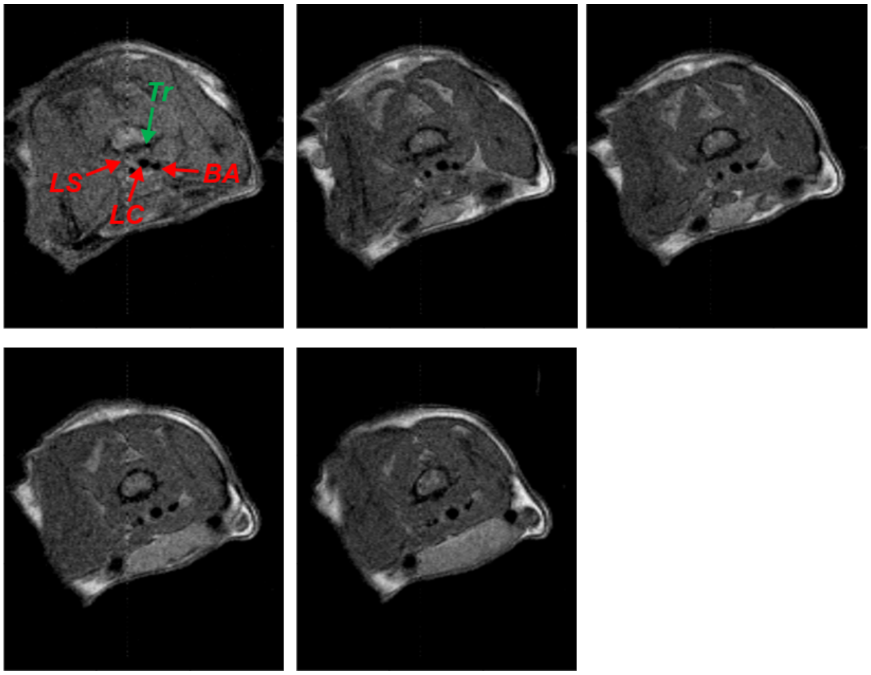

Figure S19. *In vivo* MR images of the aortic arch of an $LDLr^{-/-}$ mouse ($LDLr^{-/-}$-**E**), before **sLDL-Gd** injection. The three branches of the aortic arch and the aorta were identified by red arrows: BA = brachiocephalic artery; LC = left carotid artery; LS = left subclavian artery. The trachea is identified by green arrows. Yellow arrows point to contrast enhancement of atheroplaque in the BA wall. The three branches of the aortic arch and the aorta were identified by red arrows: BA = brachiocephalic artery; LC = left carotid artery; LS = left subclavian artery. The trachea is identified by a green arrow.

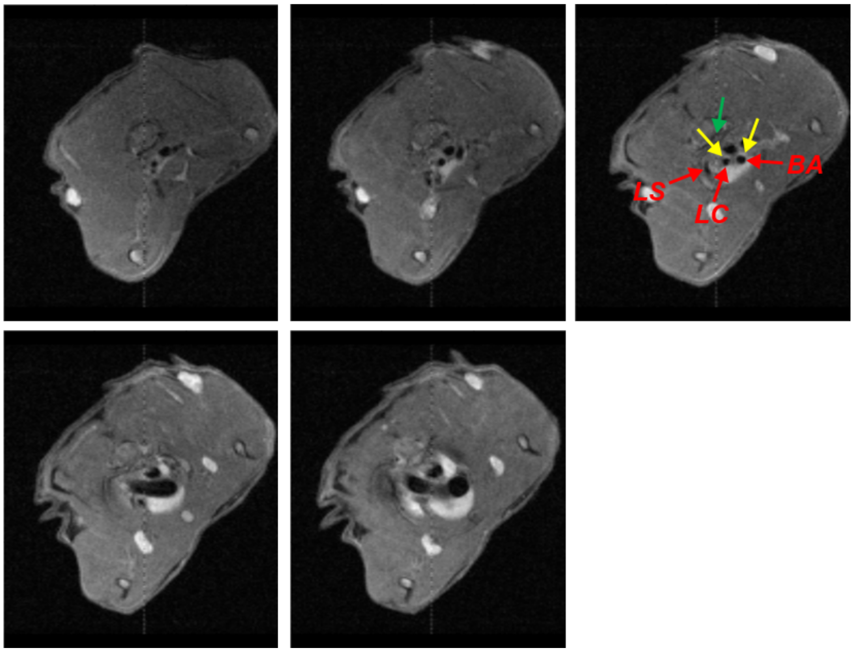

Figure S20. *In vivo* MR images of the aortic arch of the same $LDLr^{-/-}$ mouse ($LDLr^{-/-}$-**E**), at 48 h after **sLDL-Gd** injection. The three branches of the aortic arch and the aorta were identified by red arrows: BA = brachiocephalic artery; LC = left carotid artery; LS = left subclavian artery. The trachea is identified by a green arrow. Yellow arrows point to contrast enhancement of atheroplaques.

5.2 *Ex vivo* analysis of the aorta of *apoE*$^{-/-}$ and *LDLr*$^{-/-}$ mice by ICP-MS

The mice were euthanized 48 h after the injection of **sLDL-Gd** or **sLDL-GdR**. To obtain the clean aorta from the *apoE*$^{-/-}$ or *LDLr*$^{-/-}$ mice, pH 7.4 PBS(–) was perfused in the blood stream from the left ventricle of the heart and the right atrium was gently cut in order to absorb the perfusion liquid by sterile gauzes. The aorta was then dissected utilizing a dissecting microscope and the aortic arch was collected. Under a dissection microscope, the presence of atherosclerotic plaques was clearly visible. The ICP-MS analysis of the Gd contents in the mice aorta (ng of Gd^{3+} per mg of dry tissue) was carried out in Pennsylvania Animal Diagnostic Laboratory System in New Bolton Center (Kennett Square, PA, USA).

5.3 *Ex vivo* analysis by cryoViz brightfield and fluorescence imaging of *apoE*$^{-/-}$ mice

Ex vivo analysis by cryoViz was carried out on two *apoE*$^{-/-}$ mice. The mice were euthanized 24 h after the injection of **sLDL-GdR**. Immediately after euthanasia by inhalation of carbon dioxide, the mice were covered with Tissue-Tek® Optimal Cutting Temperature (O. C. T.) solution (Sakura Finetek USA, Inc., Torrance, CA, USA). This step ensures that the carcass is wet and no air bubbles are formed in the next step of embedding. The mouse is then embedded in O. C. T. inside an aluminum foil mold. The entire mold is snap-frozen in liquid nitrogen and transferred to BioInVision (Cleveland, OH, USA) for the concomitant cryosectioning and imaging using an automated microtome-blockface episcopic imaging system (CryoViz™, BioInVision) that allows for microscopic, three-dimensional resolution of fluorophores in macroscopic specimens. A 40 µm section thickness was used for whole mouse.[5]

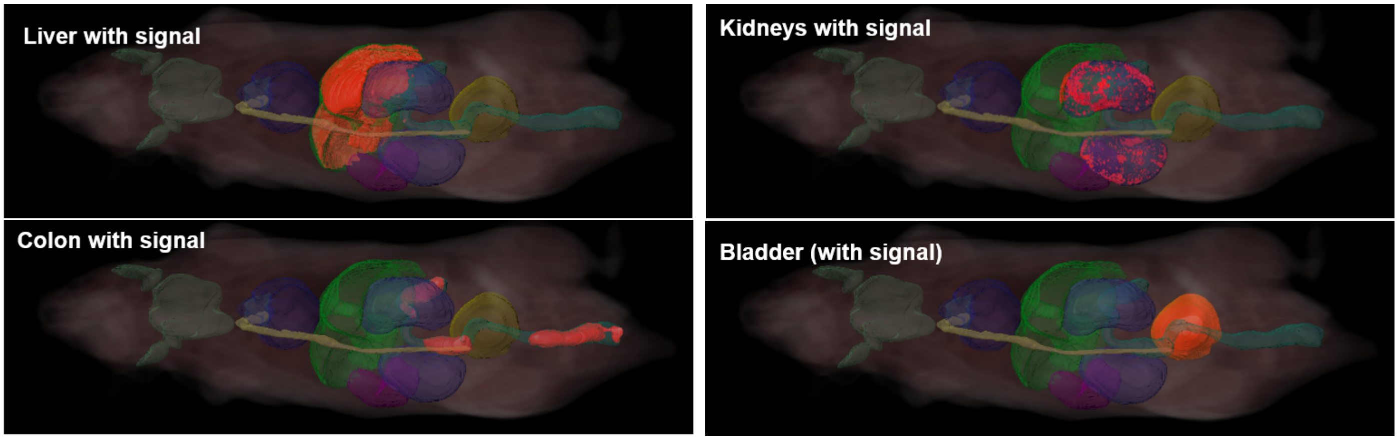

Figure S21. CryoViz 3D rendering of an *apoE*$^{-/-}$ mouse (*apoE*$^{-/-}$**-G**) ectioned along the coronal plane, showing the fluorescence signal caused by the presence of rhodamine B in liver, kidneys, colon, and bladder.

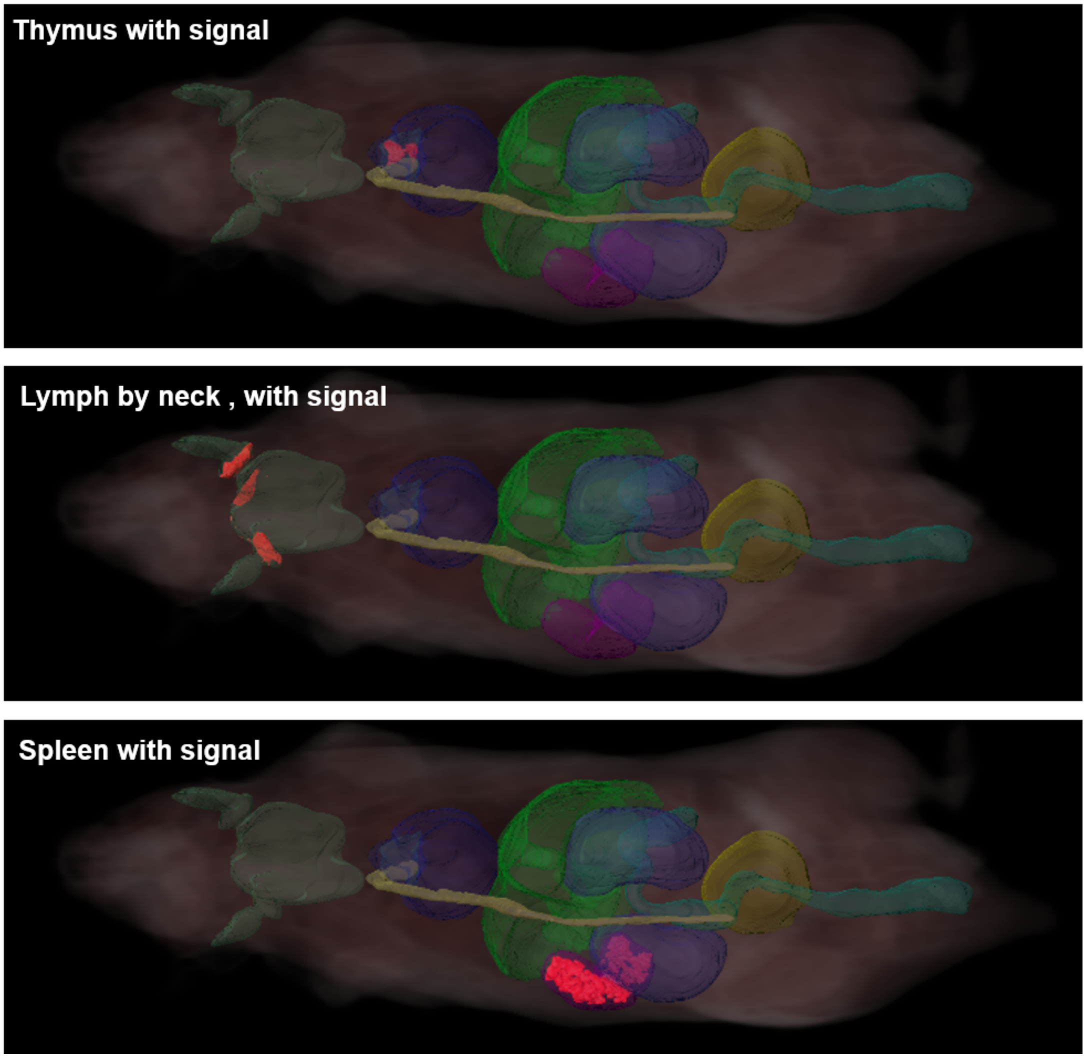

Figure S22. CryoViz 3D rendering of an *apoE*$^{-/-}$ mouse (*apoE*$^{-/-}$-**G**) sectioned along the coronal plane, showing the fluorescence signal caused by the presence of rhodamine B in thymus (top), lymph nodes (middle), spleen (bottom).

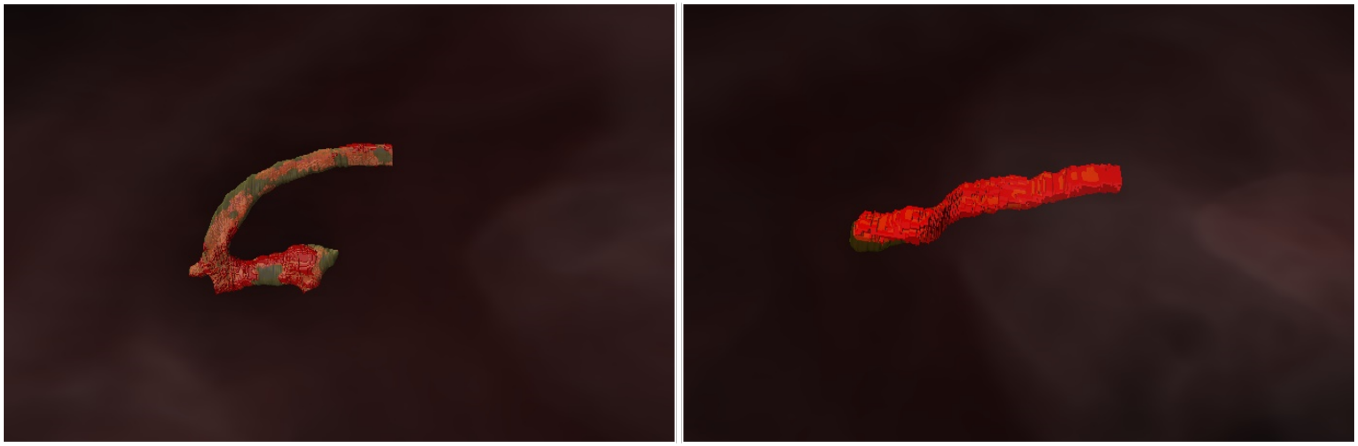

Figure S23. CryoViz 3D rendering of the aorta of an *apoE*$^{-/-}$ mouse (*apoE*$^{-/-}$-**G**) sectioned along the axial plane, showing the fluorescence signal caused by the presence of rhodamine B in ascending aorta (left), and descending aorta (right).

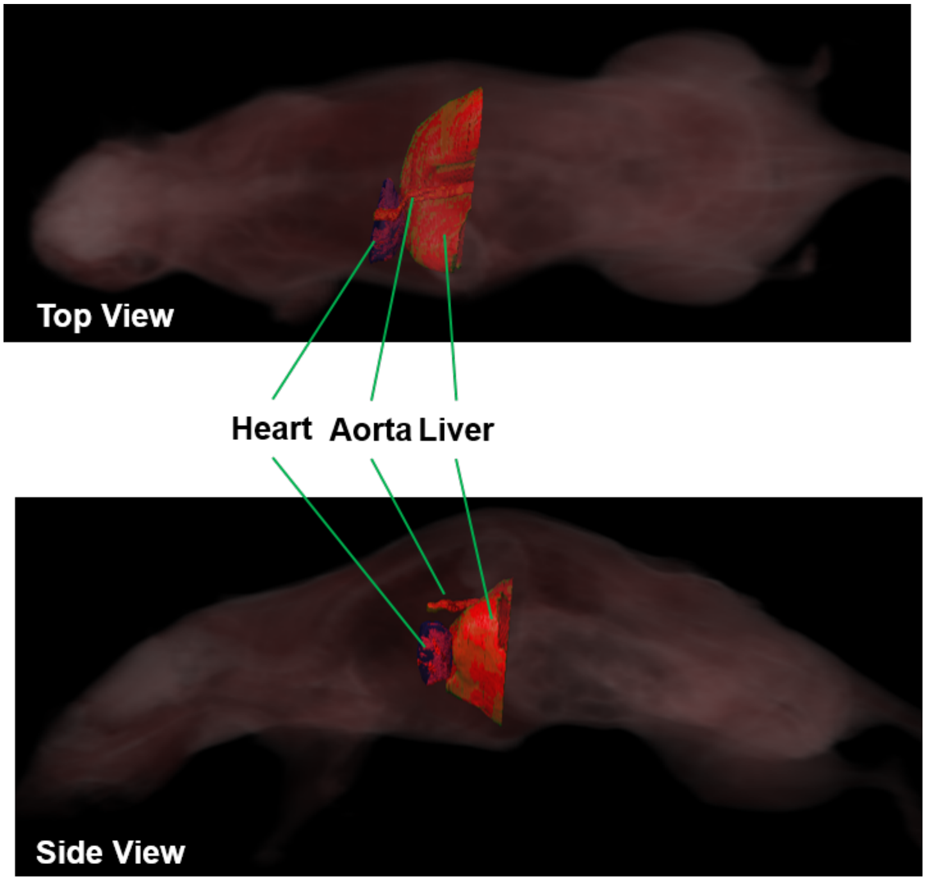

Figure S24. CryoViz 3D rendering of the aorta of an *apoE*$^{-/-}$ mouse (*apoE*$^{-/-}$**-G**) sectioned along the axial plane, showing the fluorescence signal caused by the presence of rhodamine B in the heart, aorta and liver.

6. *In vitro* cellular incorporation test with sLDL-F and LNP-F

In vitro cellular incorporation tests were performed using two types of cell lines, THP-1 (human monocyte cell line) and RAW 264.7 (mouse macrophage cell line, a gift from Dr. Atsushi Irie in Tokyo Metropolitan Institute of Medical Science). THP-1 cells were cultured in RPMI (11875 Gibco) with 10% heat-inactivated FBS(–) and 0.1 mM 2-mercaptoethanol. RAW 264.7 cells were cultured in DMEM (D5796, Sigma-Aldrich) with 10% heat-inactivated FBS and 1 mM sodium pyruvate. Cells were pre-cultured in a 6-cm dish, inoculated in the 96-well microplates with 1-4 x 10^4 cells/well. Cells were incubated for 1 or 2 days and **sLDL-F** or **LNP-F** particles were added at 5% volume of medium. to the cells in logarithmic growth phase. As a negative control, pH 7.4 PBS(–) was added at the same ratio. RAW 264.7 cells were cultured and processed in Biosafety level 2 facility.

Assay with THP-1. After the co-incubation with each particle for 5 or 20 h, cells were washed with pH 7.4

PBS(–). After removing the excess particles in the medium, cells were washed with pH 7.4 PBS(–) and incorporated particles were examined by KEYENCE Fluorescence Microscope BZ-X700 (KEYENCE Co., Osaka, Japan) using 470 nm of excitation wavelength. Phase contrast images were simultaneously recorded. The incorporated cellular fluorescent intensities of the microscopy images at ×20 magnification with ×3 digital zoom were quantified by Lumina Vision software 3.3 (Mitani Corporation, Fukui, Japan). To evaluate the difference of incorporated nanoparticles of **sLDL-F** and **LNP-F**, the fluorescence intensities per cell were evaluated by unpaired two-tailed Student's *t*-test. In Figure 7, quantified signal intensities of images and *p*-values were shown.

Assay with RAW 264.7. Cells were co-incubated with each particle for 5 h and washed with pH 7.4 PBS(–) before the examination by fluorescence microscope. Subsequently, cells were incubated in the medium without particles for additional 16 h. After washing with pH 7.4 PBS(–), the incorporated particles were examined as above. The quantification and evaluation of incorporated signals were analyzed as above.

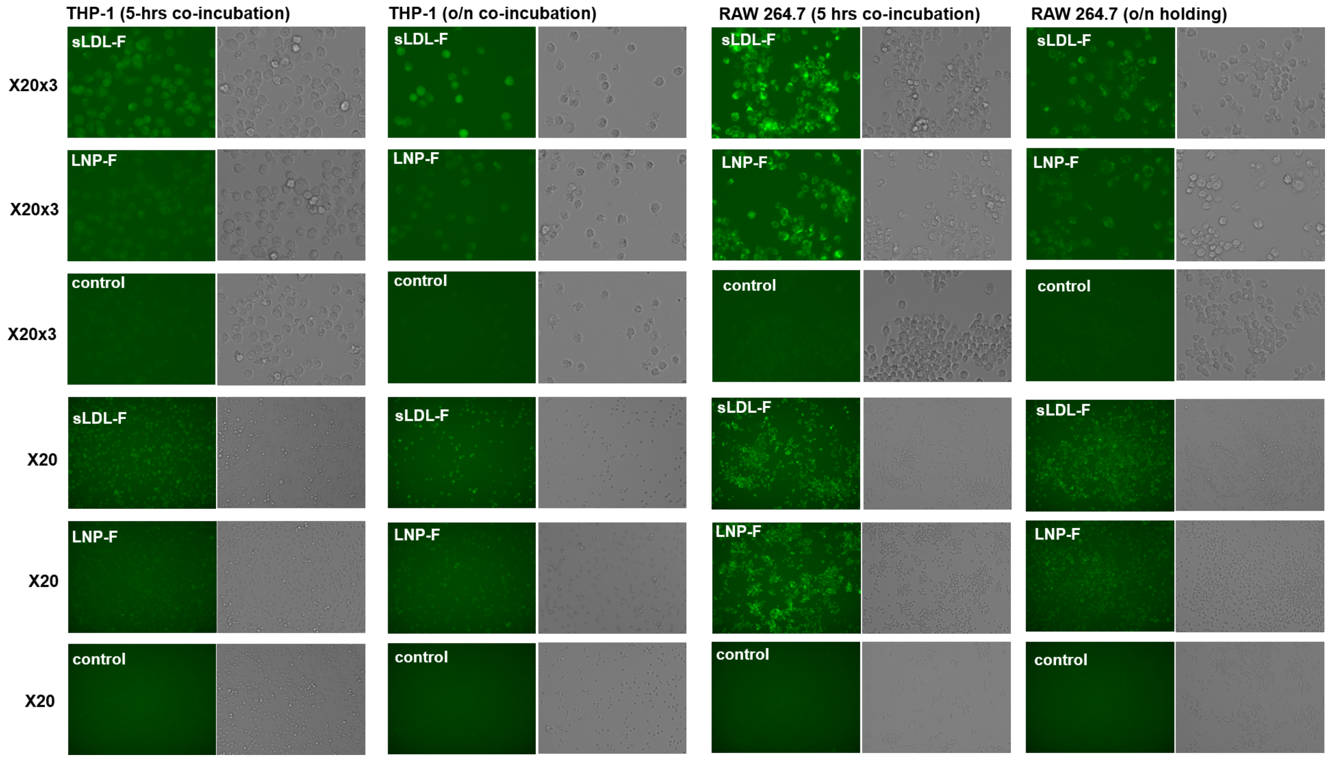

Figure S25. *In vitro* cellular incorporation and holding assay of **sLDL-F** and **LNP-F** particles. THP-1 cells were co-incubated with nanoparticles for 5 h or overnight. RAW 264.7 cells were co-incubated with nanoparticles for 5 h, washed with pH 7.4 PBS(–) and incubated overnight in the corresponding media.

References

1. Lowell, A. N.; Qiao, H.; Liu, T.; Ishikawa, T.; Zhang, H.; Oriana, S.; Wang, M.; Ricciotti, E.; FitzGerald, G. A.; Zhou, R.; Yamakoshi, Y., Functionalized Low-density Lipoprotein Nanoparticles for in Vivo Enhancement of Atherosclerosis on Magnetic Resonance Images. *Bioconjugate Chem.* **2012**, *23*, 2313-2319.

2. Yamakoshi, Y.; Qiao, H.; Lowell, A. N.; Woods, M.; Paulose, B.; Nakao, Y.; Zhang, H.; Liu, T.; Lung-Katz, S.; Zhou, LDL-based nanoparticles for contrast enhanced MRI of atheroplaques in mouse models. *Chem. Commun.* **2011**, *47*, 8835-8837.

3. Oriana, S.; Fracassi, A. Corey, A. Yamakoshi, Y., Covalent Surface Modification of Lipid Nanoparticles by Rapid Potassium Acyltrifluoroborate Amide Ligation. *Langmuir* **2018**, *34*, 13244-13251.

4. Fracassi, A.; Cao, J. B.; Yoshizawa-Sugata, N.; Toth, E.; Archer, C.; Groninger, O.; Ricciotti, E.; Tang, S. Y.; Handschin, S.; Bourgeois, J. P.; Ray, A.; Liosi, K.; Oriana, S.; Stark, W.; Masai, H.; Zhou, R.; Yamakoshi, Y. LDL-Mimetic Lipid Nanoparticles Prepared by Surface KAT Ligation for *in vivo* MRI of Atherosclerosis. *Chem. Sci.* **2020**, *11*, 11998– 12008

5. Roy, D.; Breen, M.; Salvado, O.; Heinzel, M.; McKinley, E.; Wilson, D. Imaging System for Creating 3D Block-Face Cryo-Images of Whole Mice. In *Medical Imaging 2006: Physiology, Function, and Structure from Medical Images*; International Society for Optics and Photonics, 2006; Vol. 6143, p 61431E, Wilson, D.; Roy, D.; Steyer, G.; Gargesha, M.; Stone, M.; McKinley, E. Whole Mouse Cryo-Imaging. In *Medical Imaging 2008: Physiology, Function, and Structure from Medical Images*; International Society for Optics and Photonics, 2008; Vol. 6916, p 69161I.
